# The Heterogeneity in the Landscape of Gene Dominance in Maize is Accompanied by Unique Chromatin Environments

**DOI:** 10.1093/molbev/msac198

**Published:** 2022-09-21

**Authors:** Liangwei Yin, Gen Xu, Jinliang Yang, Meixia Zhao

**Affiliations:** Department of Biology, Miami University, Oxford, OH 45056; Department of Agronomy and Horticulture, University of Nebraska-Lincoln, Lincoln, NE 68588; Center for Plant Science Innovation, University of Nebraska-Lincoln, Lincoln, NE 68583; Department of Agronomy and Horticulture, University of Nebraska-Lincoln, Lincoln, NE 68588; Center for Plant Science Innovation, University of Nebraska-Lincoln, Lincoln, NE 68583; Department of Microbiology and Cell Science, Institute of Food and Agricultural Sciences, University of Florida, Gainesville, FL 32611

**Keywords:** genome dominance, chromatin environments, gene divergence, epigenomic features, accessible chromatin regions

## Abstract

Subgenome dominance after whole-genome duplication (WGD) has been observed in many plant species. However, the degree to which the chromatin environment affects this bias has not been explored. Here, we compared the dominant subgenome (maize1) and the recessive subgenome (maize2) with respect to patterns of sequence substitutions, genes expression, transposable element accumulation, small interfering RNAs, DNA methylation, histone modifications, and accessible chromatin regions (ACRs). Our data show that the degree of bias between subgenomes for all the measured variables does not vary significantly when both of the WGD genes are located in pericentromeric regions. Our data further indicate that the location of maize1 genes in chromosomal arms is pivotal for maize1 to maintain its dominance, but location has a less effect on maize2 homoeologs. In addition to homoeologous genes, we compared ACRs, which often harbor *cis*-regulatory elements, between the two subgenomes and demonstrate that maize1 ACRs have a higher level of chromatin accessibility, a lower level of sequence substitution, and are enriched in chromosomal arms. Furthermore, we find that a loss of maize1 ACRs near their nearby genes is associated with a reduction in purifying selection and expression of maize1 genes relative to their maize2 homoeologs. Taken together, our data suggest that chromatin environment and *cis*-regulatory elements are important determinants shaping the divergence and evolution of duplicated genes.

## Introduction

Whole-genome duplication (WGD), or polyploidy, has been an important contributor of genetic novelty throughout the evolutionary history of eukaryotes (Otto and Whitton 2000; Adams and Wendel 2005; Soltis et al. 2015). Polyploidy is particularly widespread among flowering plants, many of which have undergone several rounds of WGDs (Blanc and Wolfe 2004; Jiao et al. 2011; Soltis and Soltis 2016). Following WGD, duplicated genomes experience nonequivalent genomic changes, including chromosomal rearrangements, elimination of duplicated regions, accumulation of mutations, gene conversions and translocations, and transposon insertions (Ilic et al. 2003; Gu et al. 2005; Semon and Wolfe 2007). Because of the effect that these differences in the trajectory of the evolution of duplicated genes, much effort has been spent on the dissection of the fate of these genes after WGD. In several plant species, such as *Arabidopsis thaliana* (Thomas et al. 2006), *Brassica rapa* (Wang et al. 2011), cotton (Renny-Byfield et al. 2015), wheat (Pont et al. 2013), monkey flower (Edger et al. 2017), and maize (Schnable et al. 2011), one copy of the duplicated genes (here referred to as homoeologs) is preferentially lost from one of the subgenomes, an evolutionary process referred to as “biased fractionation” (Lockton and Gaut 2005; Freeling and Thomas 2006). In maize, only 39.4% of the original duplicated gene pairs generated from the most recent WGD are still pairs today, meaning that nearly two-thirds of the original duplicated gene pairs have lost one copy and are now present as singletons (Hufford et al. 2021). Comparisons between maize and its closely related species sorghum (*Sorghum bicolor*) suggest that single gene loss via short deletions through intrachromosomal recombination is likely the primary mechanism of fractionation (Woodhouse et al. 2010; Tang et al. 2012).

In addition to fractionation, genes in the less fractionated (dominant) subgenome are under stronger purifying selection than their homoeologs in the more fractionated (recessive) subgenome (Pophaly and Tellier 2015; Zhao et al. 2017). Genes from the dominant subgenome also tend to show higher levels of expression than their duplicated copies in the recessive subgenome, known as “gene dominance” (Flagel and Wendel 2010; Schnable et al. 2011; Cheng et al. 2012; Woodhouse et al. 2014). The model proposed to explain this pattern is that the under-expressed gene of a homoeologous pair is more likely to be deleted because it produces less protein product, contributes less to function, and therefore matters less to overall fitness (Freeling et al. 2012). Interestingly, such biased fractionation is not true for all WGD events. In the recent WGD events of poplar, banana, and soybean, homoeologous genes were equally deleted and show no bias in expression when the two subgenomes are compared (Garsmeur et al. 2014; Zhao et al. 2017). Particularly in the soybean genome, which experienced a WGD roughly at the similar time as the recent tetraploid event in maize, the two subgenomes are far less distinct than those in maize. Although no subgenome dominance was observed in soybean, individual gene pairs do differentiate in a manner similar to that observed in maize (Zhao et al. 2017). This suggests that both biased and unbiased plant species may share the same mechanism with respect to gene deletion, evolution, and expression, but this process in unbiased genomes involves differences between individual genes, rather than large blocks of genes derived from single chromosomes.

The mechanism that causes differential expression of homoeologous genes remains unclear. Both genetic and epigenetic pathways may be involved in this process. Previous research has shown that small interfering RNAs (siRNAs) as well as DNA methylation triggered by those siRNAs are associated with the reduced expression of nearby genes (Hollister et al. 2011). These siRNAs and DNA methylation often target the sequences of transposable elements (TEs). This led researchers to compare the abundance of 24 nucleotide siRNAs, the level of DNA methylation, and TE accumulation near the homoeologous genes between subgenomes. In both *B. rapa* and maize, transposon-derived 24 siRNAs were more enriched in the flanking regions of the homoeologs in the recessive subgenome, which have overall lower expression values than their counterparts in the dominant subgenome (Woodhouse et al. 2014; Cheng et al. 2016; Zhao et al. 2017). In maize, the recessive genome also has higher levels of DNA methylation in all of the three sequence contexts CG, CHG (H = A, T, or C), and especially CHH, suggesting that siRNA-trigged methylation may cause downregulation of nearby genes, which may result in the biased gene loss (Renny-Byfield et al. 2017; Zhao et al. 2017). However, these subgenome differences in siRNAs are absent in the modern cotton genome, suggesting that they are not currently the primary driver of biased gene loss and expression in this species (Renny-Byfield et al. 2015). In contrast, species of monkeyflower show clear evidence of association between expression levels and siRNAs both in recent and reconstituted polyploids (Edger et al. 2017).

In both mammals and plants, transcription factors often interact with *cis*-regulatory elements, which can serve as short- or long-range enhancers/silencers to distantly interact with their target genes (Weber et al. 2016; Schmitz et al. 2022). Examples in plants include the well-known domestication gene *teosinte branched1* and the paramutable *booster1* gene in maize, *FLOWERING LOCUS T* in Arabidopsis, and the pea plastocyanin gene in pea (Chua et al. 2003; Louwers et al. 2009; Adrian et al. 2010; Studer et al. 2011). These *cis*-regulatory elements reside within accessible chromatin regions (ACRs) that are associated with active chromatin modifications on flanking nucleosomes including histone H3 lysine 4 trimethylation (H3K4me3) and H3 acetylation (H3K9/27/56ac), low nucleosome density, and low DNA methylation (Zhang et al. 2007, 2012; Roudier et al. 2011; Lu et al. 2019; Ricci et al. 2019). In maize, a large number of ACRs and chromatin loops have been identified that link long-range interaction between putative *cis*-regulatory elements and their target genes (Li et al. 2019; Lu et al. 2019; Peng et al. 2019; Ricci et al. 2019; Sun et al. 2020). The maize genome has a much larger proportion of distal ACRs (dACRs) relative to that in smaller genomes such as that of Arabidopsis, probably because of TE insertions that separate these putative regulatory regions from genes (Lu et al. 2019). Given that these *cis*-regulatory elements can regulate gene expression, we hypothesized that these regulatory elements may also show bias between the two maize subgenomes, and if so, we sought to determine whether and how such bias impacted the evolution and expression of their target genes.

Subgenome dominance has been extensively studied in maize and in many other plant species (Thomas et al. 2006; Schnable et al. 2011; Wang et al. 2011; Pont et al. 2013; Renny-Byfield et al. 2015; Edger et al. 2017). However, few of these studies have performed genome-wide comparisons of genes located in distinct chromatin environments, which have been shown to shape the patterns of divergence and retention of WGD genes (Du et al. 2012). The maize genome is composed of regions in chromosomal arms that are relatively rich in genes that are highly recombinogenic, and pericentromeric regions that have far more TEs and far fewer recombination events (Gent et al. 2012; Zhao et al. 2021). Both of these regions have large numbers of genes, but these genes inhabit quite distinct chromatin environments. In this study, we separated the maize genome into pericentromeric regions and chromosomal arms, and performed comprehensive genomic and epigenomic comparisons between maize1 and maize2. Our data show that the location of maize1 genes in chromosomal arms is pivotal for maize1 to maintain its dominance regardless of where their maize2 homoeologs are located. Remarkably, no significant bias was detected in the majority of the measured variables between maize1 and maize2 homoeologous genes when both of them are located in pericentromeric regions, suggesting that the selective forces that shape dominance are absent in these regions. We also observed that bias in these parameters in ACRs is less pronounced in the recombination-suppressed pericentromeric regions. Our research demonstrates that chromatin environment is an important factor that may shape the bias of subgenomes in maize.

## Results

### A Higher Rate of Gene Loss of Both Maize Subgenome1 (maize1) and Maize Subgenome2 (maize2) in Pericentromeric Regions than in Chromosomal Arms

**Fig. 1.**
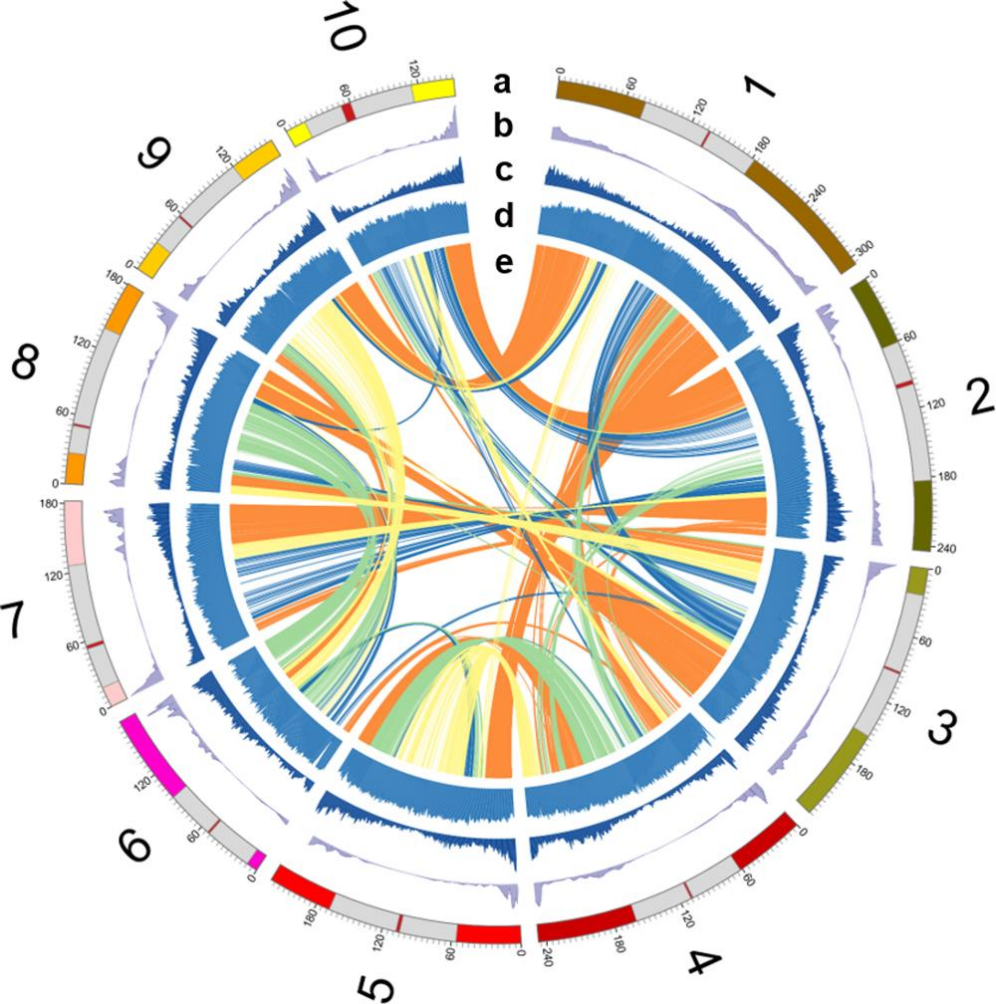
Identification of pericentromeric regions and chromosomal arms of the ten maize chromosomes. (*a*) Reference chromosomes with pericentromeric regions (near centromeres) in grey and chromosomal arms (two sides of each chromosome) in different colors for each chromosome. Presumed centromeric positions are indicated by red vertical bands (Wolfgruber et al. 2009). (*b*) Recombination rates (cM/Mb). (*c*) Gene density (number of genes/Mb). (*d*) Repeat length (Mb/Mb). (*e*) Homoeologous genes within the maize genome. Orange curves indicate both homoeologous genes in chromosome arms (M1_arm; M2_arm: 2,877 gene pairs). Blue curves indicate both homoeologous genes in pericentromeric regions (M1_peri; M2_peri: 429 gene pairs). Green curves indicate maize1 genes in chromosome arms, and maize2 genes in pericentromeric regions (M1_arm; M2_peri: 720 gene pairs). Yellow curves indicate maize1 genes in pericentromeric regions, and maize2 genes in chromosome arms (M1_peri; M2_arm: 552 gene pairs).

Previous studies have demonstrated that the chromatin environment is one of the important determinants shaping the patterns of divergence and retention of duplicated genes in rice and soybean (Tian et al. 2009; Du et al. 2012). We sought to determine whether chromatin environment also plays a role in subgenome fractionation in maize. We examined 24,616 genes in the maize genome that have syntelogs (genes with syntenic homologous relationship) in the sorghum genome (supplementary fig. S1, Supplementary Material online). Given that genes involved in tandem duplication have an ambiguous retention status, these genes were removed from future analysis. This left 4,578 syntenic duplicated gene pairs and 11,554 syntenic singletons as the final data set for further analysis (tables 1 and 2, and supplementary fig. S1, Supplementary Material online). Here and throughout, we refer to either maize1 or maize2 genes within these 4,578 duplicated gene pairs as WGD genes. To determine whether biased fractionation of the two maize subgenomes is different between chromosomal arms and pericentromeric regions, we separated the ten maize chromosomes into chromosomal arms and pericentromeric regions based on the gene and TE density as well as recombination rates (cM/Mb) determined by 6,257 genetic markers (fig. 1; Liu et al. 2009; Zhao et al. 2021). Next, we compared the distribution of the 4,578 duplicated gene pairs and 11,554 singletons in these two genomic regions. In maize1, the ratio of singletons to WGD genes (1.7:1; 5,937 singletons and 3,597 WGD genes) in chromosomal arms is significantly lower than that of singletons to WGD genes (2.2:1; 2,131 singletons and 981 WGD genes) in pericentromeric regions (supplementary table S1, Supplementary Material online, *P* < 0.0001, χ^2^ test). The same pattern was observed in maize2. The ratio of singletons to WGD genes is 0.7:1 in chromosomal arms (2,448 singletons and 3,429 WGD genes) versus 0.9:1 in pericentromeric regions (1,038 singletons and 1,149 WGD genes; supplementary table S1, Supplementary Material online, *P* < 0.0001, χ^2^ test). These data suggest that both maize1 and maize2 exhibit a higher level of gene loss in pericentromeric regions than in chromosomal arms, and this difference is larger in the dominant genome maize1 than in the recessive genome maize2 (tables 1 and 2).

**Table 1.** Comparisons of the Duplicated Blocks in the Same Chromatin Environment

Features	Maize1 in Chromosomal Arms	Maize2 in Chromosomal Arms	*P*-values	Maize1 in Pericentromeric Regions	Maize2 in Pericentromeric Regions	*P*-values
No. of blocks	46 pairs			24 pairs		
Singletons vs. WGD genes^a^	4,232 vs. 2,877	1,866 vs. 2,877	<0.0001^b^	797 vs. 429	375 vs. 429	<0.0001^b^
No. of genes	167.31 ± 205.22	86.64 ± 111.12	0.0001^c^	49.54 ± 35.93	32.13 ± 23.49	0.0001^c^
Average sizes of blocks (Mb)	10.82 ± 13.42	4.92 ± 5.48	0.0009^c^	11.87 ± 11.20	5.01 ± 4.37	0.0005^c^
Gene densities (no./Mb)	15.59 ± 5.37	17.54 ± 7.85	0.0884^c^	4.86 ± 2.37	7.35 ± 2.80	0.0013^c^
Retention rates^d^	0.76 ± 0.08	0.52 ± 0.10	<0.0001^c^	0.72 ± 0.15	0.53 ± 0.16	<0.0051^c^
TEs (DNA, Mb)	7.35 ± 9.31	3.30 ± 3.73	0.0016^c^	8.51 ± 7.96	3.58 ± 3.12	0.0004^c^
LTR-RTs (DNA, Mb)	6.10 ± 7.95	3.44 ± 3.64	0.0012^c^	7.93 ± 7.45	3.30 ± 2.89	0.0005^c^
DNA TEs (DNA, Mb)	0.64 ± 0.75	0.37 ± 0.38	0.0001^c^	0.56 ± 0.51	0.28 ± 0.24	0.0003^c^
Recombination rates (cM/Mb)^e^	2.47 ± 1.60	3.19 ± 2.75	<0.0001^c^	0.34 ± 0.25	0.37 ± 0.26	0.0724^c^

LTR-RTs, long terminal repeat retrotransposons; TEs, transposable elements; WGD, whole-genome duplication.

a Genes in maize that show a syntenic relationship with the genes in sorghum. Genes involved in tandem duplication were not included here.

b χ^2^ test.

c Student’s paired *t*-test.

d Retention rates were calculated based on the retained genes out of the total ancestral genes. For example, for each block, maize1 retention rate = (maize1 singletons + maize1 WGD genes)/(maize1 singletons + maize2 singletons + maize1 WGD genes).

e Recombination rates were compared based on the duplicated gene pairs.

**Table 2.** Comparisons of the Duplicated Blocks in Different Chromatin Environments

Features	Maize1 in Chromosomal Arms	Maize2 in Pericentromeric Regions	*P*-values	Maize1 in Pericentromeric Regions	Maize2 in Chromosomal Arms	*P*-values
No. of blocks	32 pairs			17 pairs		
Singletons vs. WGD genes^a^	1,536 vs. 720	649 vs. 720	<0.0001^b^	1,372 vs. 552	570 vs. 552	<0.0001^b^
No. of genes	71.88 ± 92.19	43.91 ± 56.01	0.0003^c^	118.71 ± 116.74	72.24 ± 79.12	<0.0001^c^
Average sizes of blocks (Mb)	5.23 ± 6.66	6.91 ± 10.23	0.1303^c^	19.59 ± 16.03	6.26 ± 6.22	0.0002^c^
Gene densities (no./Mb)	14.15 ± 6.96	8.24 ± 5.81	<0.0007^c^	6.90 ± 2.56	12.04 ± 5.07	0.0007^c^
Retention rates^d^	0.76 ± 0.15	0.47 ± 0.16	<0.0001^c^	0.79 ± 0.08	0.43 ± 0.11	<0.0001^c^
TEs (DNA, Mb)	3.60 ± 4.75	4.96 ± 7.34	0.0943^c^	13.91 ± 11.28	4.32 ± 4.35	0.0002^c^
LTR-RTs (DNA, Mb)	3.26 ± 4.36	4.60 ± 6.86	0.0830^c^	12.85 ± 10.48	3.89 ± 3.92	0.0002^c^
DNA TEs (DNA, Mb)	0.32 ± 0.38	0.35 ± 0.47	0.5146^c^	1.03 ± 0.82	0.40 ± 0.41	<0.0001^c^
Recombination rates (cM/Mb)^e^	2.81 ± 2.70	0.49 ± 0.45	<0.0001^c^	0.34 ± 0.23	2.30 ± 2.34	<0.0001^c^

LTR-RTs, long terminal repeat retrotransposons; TEs, transposable elements; WGD, whole-genome duplication

a Genes in maize that show a syntenic relationship with the genes in sorghum. Genes involved in tandem duplication were not included here.

b χ^2^ test.

c Student’s paired *t*-test.

d Retention rates were calculated based on the retained genes out of the total ancestral genes. For example, for each block, maize1 retention rate = (maize1 singletons + maize1 WGD genes)/(maize1 singletons + maize2 singletons + maize1 WGD genes).

e Recombination rates were compared based on the duplicated gene pairs.

To further examine the effects of chromatin environment on the retention of duplicated genes, maize’s 10 chromosomes (v4) were split into 119 duplicated block pairs. Of the 119 duplicated block pairs, 46 (38.7%), including 11,852 genes, are with both blocks in chromosomal arms (M1-arm vs. M2-arm), and 24 (20.2%), including 2,030 genes, are with both blocks in pericentromeric regions (M1-peri vs. M2-peri; fig. 1, table 1, and supplementary fig. S2, Supplementary Material online). Thus, in only 59% of blocks, both members of each pair are in the same chromatin environment, and 41% of the blocks have divergent chromatin characteristics. Because maize is an allotetraploid, these differences could have been present prior to polyploidy around 12 Ma or could have occurred after it. In either event, these divergent blocks amount to a natural experiment in which duplicated homoeologs are placed into distinct chromatin environments. A total of 32 (26.9%) of these divergent blocks, including 3,625 genes, have maize1 in chromosomal arms and maize2 in pericentromeric regions (M1-arm vs. M2-peri), and 17 (14.3%) duplicated blocks, including 3,046 genes, are with maize1 in pericentromeric regions and maize2 in chromosomal arms (M1-peri vs. M2-arm; fig. 1, table 2, and supplementary fig. S2, Supplementary Material online). When the blocks of the two subgenomes are in the same chromatin environment (M1-arm vs. M2-arm and M1-peri vs. M2-peri), maize1 blocks are generally larger, have more genes, more TEs, lower gene densities, and lower recombination rates (cM/Mb; table 1). Interestingly, we find that the difference of the recombination rates between the two subgenomes are less pronounced when both maize1 and maize2 blocks are in pericentromeric regions than when both are in chromosomal arms (table 1). In contrast, when the blocks of the two subgenomes are in different chromatin environments (M1-arm vs. M2-peri and M1-peri vs. M2-arm), the subgenome blocks that are in pericentromeric regions are generally larger, and have more TEs, lower gene densities, and lower recombination rates regardless of the subgenome (table 2).

### Stronger Purifying Selection of Maize1 Over Maize2 is Less Pronounced in Pericentromeric Regions

**Fig. 2.**
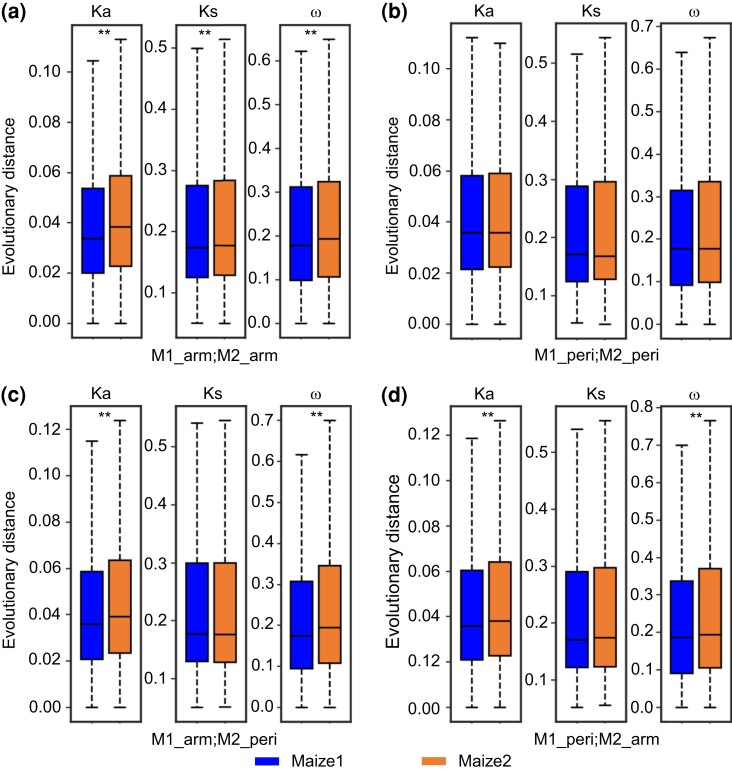
Stronger purifying selection of maize1 over maize2 is less pronounced in pericentromeric regions. (*a*) Both homoeologous genes in chromosome arms (M1_arm; M2_arm: 2,877 gene pairs). (*b*) Both homoeologous genes in pericentromeric regions (M1_peri; M2_peri: 429 gene pairs). (*c*) Maize1 genes in chromosome arms, and maize2 genes in pericentromeric regions (M1_arm; M2_peri: 720 gene pairs). (*d*) Maize1 genes in pericentromeric regions, and maize2 genes in chromosome arms (M1_peri; M2_arm: 552 gene pairs). The statistical analysis was conducted using Student’s paired *t*-test. *P < 0.05; **P < 0.01.

Previous research has demonstrated that maize1 genes are under stronger purifying selection than are maize2 genes (Pophaly and Tellier 2015; Zhao et al. 2017). We next asked whether this difference in purifying selection is associated with the differences in chromatin environment. To do this, we compared the evolutionary distances of the 4,578 duplicated gene pairs in the four categories described above. When both homoeologous genes are in chromosomal arms (M1-arm vs. M2-arm), Ka (nonsynonymous substitution), Ks (synonymous substitution), and ω (Ka/Ks) of maize1 genes are all significantly lower than those of their maize2 homoeologs, indicating that maize1 has experienced an overall lower mutation rate as well as a higher level of purifying selection relative to maize2 (fig. 2*a*), as has been noted previously (Pophaly and Tellier 2015; Zhao et al. 2017). In the M1-arm versus M2-peri and M1-peri versus M2-arm categories, both Ka and ω were significantly lower for maize1 than for maize2, also consistent with relaxed selection on maize2 genes (fig. 2*c*and*d*). In contrast, when both homoeologous genes are in pericentromeric regions (M1-peri vs. M2-peri), we find no significant differences in Ka, Ks, or ω between maize homoeologs (fig. 2*b*).

Next, we compared the numbers of putatively deleterious alleles (the genetic load) between maize1 and maize2 using genomic evolutionary rate profiling (GERP) scores (Rodgers-Melnick et al. 2015; Wang et al. 2017; Yang et al. 2017). GERP score, a measure of sequence conservation across the phylogeny (Huber et al. 2020), estimates purifying selection in terms of rejected substitutions relative to the neutral expectation. Scores >0 may reflect purifying selection, and mutations at such sites are more likely to be deleterious. In this study, we only retained nonsynonymous single-nucleotide polymorphism (SNP) sites with GERP scores >0 as putatively deleterious sites. Our data show a significant higher number of putatively deleterious alleles in maize2 genes than in maize1 genes when they are both located in chromosomal arms. In contrast, no significant difference was observed with respect to the genetic load between maize1 and maize2 genes when they are located in pericentromeric regions (supplementary fig. S3, Supplementary Material online). These results echo the ω analysis, indicating that the difference in purifying selection between homoeologs is ameliorated when both homoeologs are in pericentromeric regions.

We also compared Ka, Ks, and ω of genes in chromosomal arms with those genes in pericentromeric regions. For both maize1 and maize2, the average Ks and recombination rates of WGD genes in pericentromeric regions are significantly lower than those in chromosomal arms (supplementary fig. S4, Supplementary Material online). In contrast, there was no significant difference in Ka, gene expression or protein abundance for either maize1 or maize2 when comparing WGD genes in these two chromatin environments. Finally, we compared the evolutionary distances between WGD genes and singletons. Overall, for both maize1 and maize2, and for both pericentromeric regions and chromosomal arms, the average Ka for WGD genes is significantly lower than that for singletons, whereas no significant difference in Ks was detected between WGD genes and singletons (supplementary figs. S5 and S6, Supplementary Material online). This difference between WGD genes and singletons is associated with expression differences between these two classes of genes. On average, singletons are expressed at a significantly lower level than WGD genes (supplementary figs. S5 and S6, Supplementary Material online), suggesting duplication status is an important factor contributing to gene evolution and function.

### Biased Expression Between Homoeologs of Maize is Weaker in Pericentromeric Regions

**Fig. 3.**
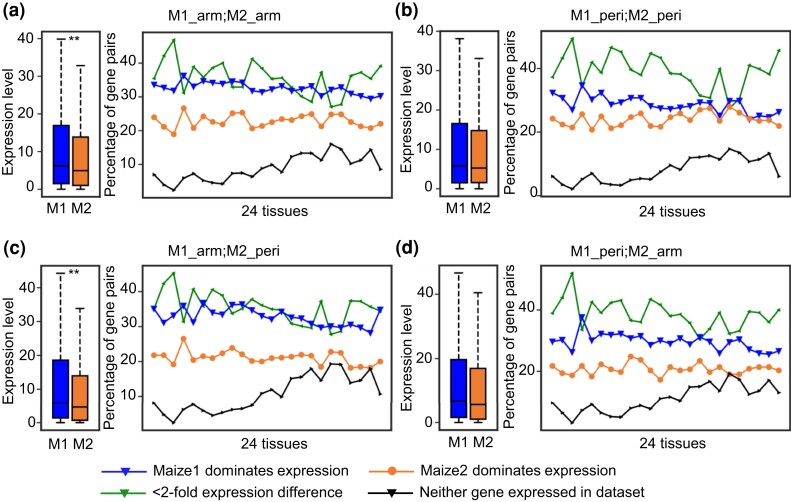
Biased expression between two homoeologs of maize is weaker in pericentromeric regions. (*a*) Both homoeologous genes in chromosome arms (M1_arm; M2_arm: 2,877 gene pairs). (*b*) Both homoeologous genes in pericentromeric regions (M1_peri; M2_peri: 429 gene pairs). (*c*) Maize1 genes in chromosome arms, and maize2 genes in pericentromeric regions (M1_arm; M2_peri: 720 gene pairs). (*d*) Maize1 genes in pericentromeric regions, and maize2 genes in chromosome arms (M1_peri; M2_arm: 552 gene pairs). The left boxplot of each panel represents the overall expression values (FPKM, fragments per kilobase of exon per million mapped fragments) of the homoeologous genes from 24 tissues, and the right plot indicates expression pattern of the duplicated gene pairs following the horse race method previously described (Schnable et al. 2011; Woodhouse et al. 2014; Zhao et al. 2017). RNA-seq data of the 24 tissues were from (Sekhon et al. 2013; Eveland et al. 2014). These tissues include germinating seeds (24 h after germination), primary root (6 days after sowing), shoot apical meristem (vegetative 3), leaf tip (vegetative 5), vascular leaf (vegetative 9, immature), vascular leaf (vegetative 9, eighth leaf), vascular leaf (vegetative 9, 11th leaf), vascular leaf (vegetative 9, 13th leaf), vascular leaf (vegetative tasseling, 13th leaf), vascular leaf (reproductive 2, 13th leaf), 10 DAP (days after pollination) whole seed, 12 DAP whole seed, 14 DAP whole seed, 16 DAP whole seed, 12 DAP endosperm, 14 DAP endosperm, 16 DAP endosperm, 16 DAP embryo, ear tip, ear mid, ear base, tassel stage 1, tassel stage 2, and tassel stage 3. The statistical analysis was conducted using Student’s paired *t*-test. **P < 0.01.

To understand the functional divergence of maize homoeologous genes, we measured gene expression using publicly available RNA-seq data from 24 maize tissues (Sekhon et al. 2013; Eveland et al. 2014). In each tissue, we compared the number of duplicated gene pairs in which either maize1 or maize2 dominated expression following the method described in previous studies (Schnable et al. 2011; Woodhouse et al. 2014; Zhao et al. 2017). Dominant expression was defined as instances in which expression of one homoeolog is two-fold or greater than the expression of the other homoeolog in that tissue. In all four categories, we observed a bias towards gene pairs dominated by expression of the maize1 copy, regardless of their chromatin environment (fig. 3). For instance, when both maize1 and maze2 are in chromosomal arms, on average 32.5% maize1 WGD genes dominate expression, which is 9.6% higher than the average percentage (22.9%) of their maize2 homoeologous genes dominating expression in the 24 tissues (fig. 3*a*). However, this difference of dominance between maize1 and maize2 is smaller when the two homoeologs are both in pericentromeric regions (28.7% vs. 24%). It is worth noting that in some tissues such as base of the ear, no or marginal biased expression between homoeologs was observed when both are in pericentromeric regions (fig. 3*b*). The expression differences between maize1 and maize2 are even larger when maize1 genes are in chromosomal arms and their maize2 homoeologs are in pericentromeric regions (32.8% vs. 21.1%; fig. 3*c*). In addition, we examined the average expression values (FPKM, fragments per kilobase of exon per million mapped fragments) of the duplicated gene pairs in the four categories. The average expression level of maize1 was observed to be significantly higher than that of maize2 only when maize1 genes are in chromosomal arms regardless of where their maize2 homoeologs are located (fig. 3*a*and*c*, left panels). We find no significant differences in mean expression between homoeologs when maize1 genes are in pericentromeric regions (fig. 3*b*and*d*, left panels).

Next, we analyzed the protein abundance using publicly available data from 148 samples of 23 tissues with the same cutoff as that for RNA expression (Walley et al. 2016; Walsh et al. 2020). In all the four categories, maize1 dominates protein abundance in most of the samples (supplementary fig. S7, Supplementary Material online). However, the difference of the biased protein abundance is smaller than that of the biased RNA expression (fig. 3 and supplementary fig. S7, Supplementary Material online). Overall, our data demonstrate that the dominance of maize1 is at both transcriptional and translational levels and differs in different genomic regions.

### TEs and Their Associated Epigenetic Marks have Shaped the Two Subgenomes in Different Chromatin Environments

**Fig. 4.**
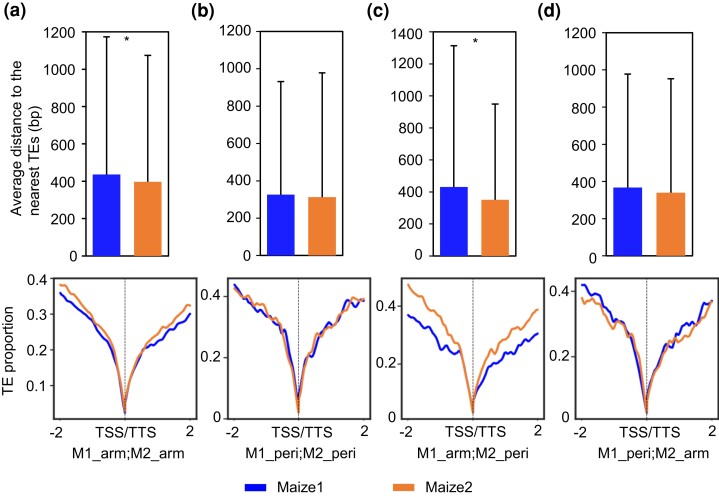
No significant differences in transposable elements flanking homoeologous genes when maize1 is in pericentromeric regions. (*a*) Both homoeologous genes in chromosome arms (M1_arm; M2_arm: 2,877 gene pairs). (*b*) Both homoeologous genes in pericentromeric regions (M1_peri; M2_peri: 429 gene pairs). (*c*) Maize1 genes in chromosome arms, and maize2 genes in pericentromeric regions (M1_arm; M2_peri: 720 gene pairs). (*d*) Maize1 genes in pericentromeric regions, and maize2 genes in chromosome arms (M1_peri; M2_arm: 552 gene pairs). The top figure of each panel indicates the average distance of WGD genes to their nearest TEs, and the bottom figure represents the TE proportions in the 2 kb upstream and downstream regions of WGD genes. These regions were divided into 100 bp sliding windows with 10 bp increments. TSS, transcription start site; TTS, transcription termination site. The statistical analysis was conducted using Student’s paired *t-*test. *, *P* < 0.05.

Given that silenced TEs have deleterious effects on neighboring gene expression (Hollister and Gaut 2009), we wondered whether TEs were associated with differences in expression patterns of WGD genes in different genomic locations. We compared the abundance of flanking TEs and their distances to nearby WGD genes located in either chromosomal arms or pericentromeric regions. Previously maize1 genes were found to be significantly farther from TEs than are their maize2 homoeologs (Zhao et al. 2017). Interestingly, our data show that this is only the case when maize1 genes are in chromosomal arms, regardless of the location of their maize2 homoeologs (fig. 4*a*and*c*, top panels). In contrast, no significant differences with respect to the distances to TEs were observed when maize1 genes are in pericentromeric regions (fig. 4*b*and*d*, top panels). We also measured the TE abundance in the 2 kb upstream and downstream regions of duplicated gene pairs. The abundance of TEs around maize1 genes is obviously lower than those of TEs around their maize2 homoeologs only when maize1 genes are in chromosomal arms (fig. 4*a*and*c*, bottom panels). Although the location of maize2 genes is not as important, the differences with respect to TE distances to nearest genes and TE abundances between maize1 and maize2 are larger when maize2 homoeologs are in pericentromeric regions (fig. 4*a*and*c*). These data are consistent with previous hypotheses that purifying selection more efficiently purges TEs near maize1 genes and suggests that this process is more efficient when these genes are in chromosomal arms.

**Fig. 5.**
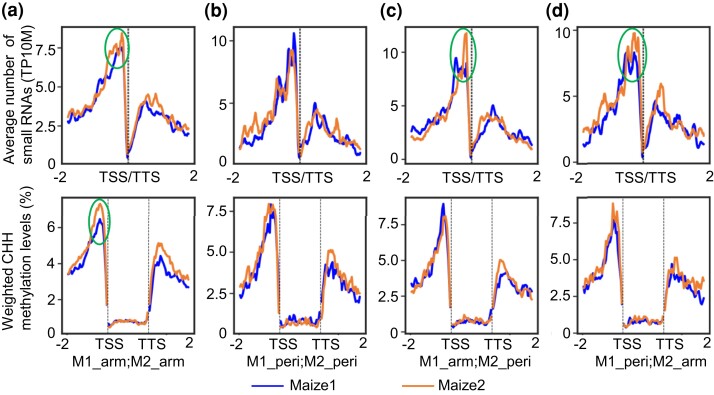
Distribution and abundances of 24 nucleotide small RNAs and CHH (H = A, T, or C) methylation around and on homoeologous genes in different chromatin environments. (*a*) Both homoeologous genes in chromosome arms (M1_arm; M2_arm: 2,877 gene pairs). (*b*) Both homoeologous genes in pericentromeric regions (M1_peri; M2_peri: 429 gene pairs). (*c*) Maize1 genes in chromosome arms, and maize2 genes in pericentromeric regions (M1_arm; M2_peri: 720 gene pairs). (*d*) Maize1 genes in pericentromeric regions, and maize2 genes in chromosome arms (M1_peri; M2_arm: 552 gene pairs). Both methylation levels and small RNA abundance were averaged from different maize tissues. Only uniquely and perfectly mapped 24 nt small RNAs were averaged in a 100 bp sliding window moving in 10 bp increments of the 2 kb upstream and downstream regions of the duplicated gene pairs following the methods previously described (Woodhouse et al. 2014; Zhao et al. 2017). Weighted CHH methylation levels were calculated in a 50 bp window. Gene body methylation was measured on 40 equally sized bins. Bin sizes differ from gene to gene because of the different lengths of genes. TP10M, transcripts per 10 million uniquely and perfectly mapped reads; TSS, transcription start site; TTS, transcription termination site.

Compared with chromosomal arms, recombination-suppressed pericentromeric regions are associated with a lower abundance of 24 nt siRNAs, a higher level of CG and CHG DNA methylation, and enriched repressive histone modifications (Gent et al. 2012). Given that biased evolution and expression between homoeologous genes were observed to be less pronounced in pericentromeric regions (figs. 2 and 3), we asked whether these epigenetic marks are more generally associated with the reduced evolution and expression. Small RNAs from four maize tissues were perfectly and uniquely mapped to the reference genome B73 (version 4; Bousios et al. 2016; Jiao et al. 2017), and were evaluated for abundance and distribution around the 2 kb upstream and downstream regions of the duplicated gene pairs in the four categories described above. Consistent with previous results (Zhao et al. 2017), 24 nt siRNAs around the duplicated gene pairs are distributed into two peaks, and the peaks around maize1 genes are lower than those around maize2 genes (fig. 5, top panels, and supplementary fig. S8, Supplementary Material online). When different categories are compared, maize1 genes are targeted less by 24 nt siRNAs than are maize2 genes in three of the four categories (M1-arm vs. M2-arm, M1-arm vs. M2-peri, and M1-peri vs. M2-arm). In contrast, no such difference was observed between maize1 and maize2 homoeologous genes when both are in pericentromeric regions (fig. 5*b*, top panel).

Given that 24 nt siRNAs can recruit histone modifiers and DNA methyltransferases to trigger methylation of DNA (Matzke and Mosher 2014; Matzke et al. 2015), we next asked whether the levels of DNA methylation mediated by siRNAs are different between maize1 and maize2 homoeologs in different genomic regions. As symmetrical methylation CG and CHG can be maintained independently of siRNAs during DNA replication, but asymmetrical CHH methylation requires siRNA trigger, we specially focus on cytosine methylation in this sequence context. As is the case for 24 nt siRNAs, CHH methylation has two peaks, previously designated CHH islands (Gent et al. 2013; Li et al. 2015), at the roughly similar positions as the small RNA peaks in the upstream and downstream regions. CHH islands in regions downstream of genes are higher in maize2 than in maize1 in all the four categories. However, CHH islands in the upstream regions are higher in maize2 only when both homoeologous genes are in chromosomal arms (fig. 5*a*, bottom panels, and supplementary figs. S9 and S10, Supplementary Material online). Together with small RNA data, our methylation data suggest that maize2 genes have a higher level of methylation adjacent to genes than do maize1 genes, particularly when two homoeologs are in chromosomal arms.

Next, we examined histone modifications between the duplicated gene pairs using previously published epigenetic data (Ricci et al. 2019; Long et al. 2021). These histone modifications include the histone modifications associated with active chromatin, H3K4me1, H3K4me3, H3K27me3, H3K36me3, H3K9ac, H3K27ac, and H3K56ac, H3K9me2 that is associated with inactive chromatin, and the histone variant H2A.Z, which can be associated with active and inactive chromatin depending on its location within the gene body (supplementary figs. S11 and S12, Supplementary Material online). We find that when the homoeologs are in the same chromatin environment (M1-arm vs. M2-arm and M1-peri vs. M2-peri), the levels of active histone modifications are all slightly higher in the 2 kb upstream and downstream regions and gene bodies of maize1 than those of maize2, and the levels of H3K9me2 is marginally lower for maize1 (supplementary figs. S11a, b, S12a and b, Supplementary Material online). Given that chromosomal arms are generally less compact than pericentromeric regions, it is not surprising that when maize1 genes are in chromosomal arms and their maize2 homoeologs are in pericentromeric regions (M1-arm vs. M2-peri), the levels of active histone modifications are much higher in the flanking regions of maize1 than those of maize2, and the level of the repressive histone modification H3K9me2 is dramatically lower for maize1 (supplementary figs. S11c and S12c, Supplementary Material online). When maize1 genes are in pericentromeric regions and their maize2 homoeologs are in chromosomal arms (M1-peri vs. M2-arm), we expected to see higher levels of active histone modifications and lower levels of repressive histone modifications in maize2 than those in maize1. However, no obvious differences were detected between maize1 and maize2 homoeologs with respect to these histone modifications (supplementary figs. S11d and S12d, Supplementary Material online), suggesting that selection against the accumulation of TEs (fig. 4), which are the major targets of repressive histone modifications, near maize2 genes was relaxed even though they are in chromosomal arms.

### Presence or Absence of ACRs may Have Affected the Biased Evolution and Expression of their Flanking WGD Genes

**Fig. 6.**
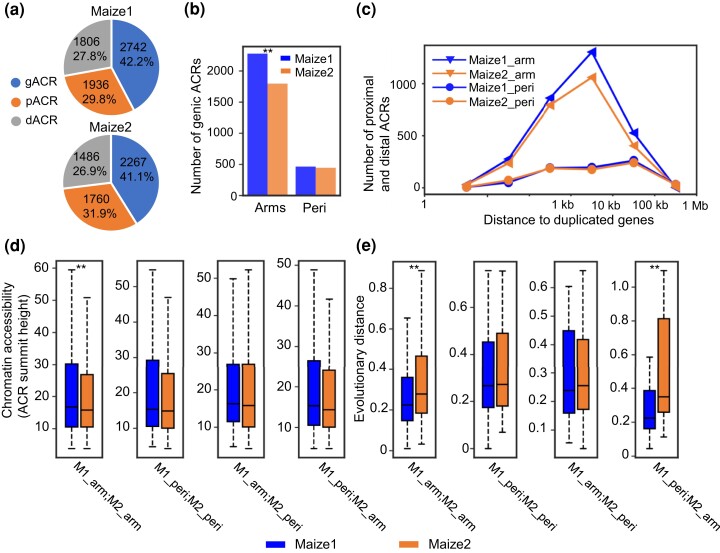
Differences in accessible chromatin regions (ACRs) between maize1 and maize2. (*a*) Numbers of genic, proximal, and distal ACRs (gACRs, pACRs, and dACRs) in maize1 and maize2. (*b*) Numbers of genic ACRs in chromosomal arms (arms) and in pericentromeric regions (peri). The statistical analysis was conducted using χ^2^ test. **P < 0.01. (*c*) Numbers of proximal and distal ACRs and their distances to nearest WGD genes. (*d*) Comparison of chromatin accessibilities of ACRs between maize1 and maize2 in different chromatin environments following the four same categories defined for homoeologous genes. Only 2,102 syntenic ACRs were used here. The statistical analysis was conducted using Student’s *t*-test. **P < 0.01. (*e*) Comparison of evolutionary distances (K) of ACRs between maize1 and maize2 in different categories. Only 381 syntenic ACR pairs (both ACRs are retained in maize1 and maize2, and have syntelogs in sorghum) were used here. The statistical analysis was conducted using Student’s paired *t*-test. **P < 0.01.

In mammalian and plant genomes, *cis*-regulatory elements that reside within accessible chromatin have been found to interact with their target genes to regulate their expression (Thurman et al. 2012; Shlyueva et al. 2014; Weber et al. 2016; Lu et al. 2019; Ricci et al. 2019; Schmitz et al. 2022). Particularly, such interaction between transcription factors, regulatory sequences and genes often disrupts nucleosome formation, which results in ACRs that harbor putative *cis*-regulatory elements (Iwafuchi-Doi et al. 2016; Klemm et al. 2019). Given the biased expression of the maize homoeologs, we sought to determine whether ACRs have been biased fractionated between maize1 and maize2 and whether they have shaped the expression patterns of the homoeologous genes. In order to do so, we reanalyzed 32,111 publicly available ACRs (Ricci et al. 2019). Based on their distances to their nearest annotated genes, we find that 11,997 ACRs are near WGD genes and 11,479 ACRs are near singleton genes (supplementary fig. S13, Supplementary Material online). It is worth noting that the remaining 8,635 ACRs were removed in this analysis given that their nearest genes do not have syntenic relationship with genes in sorghum. Next, we split these ACRs into the two subgenomes. The ratio of ACRs that are near singletons to ACRs that are near WGD genes in maize1 (1.3:1; 8,523 to 6,484) is significantly higher than that of ACRs near singletons to ACRs near WGD genes in maize2 (0.5:1; 2,956 to 5,513; *P* < 0.0001, χ^2^ test), suggesting ACRs are more retained in maize1. This shows that not only are maize2 genes more likely to be fractionated, but even when they are retained, their ACRs are more likely to be fractionated. The distribution of the position of the ACRs relative to the genes was similar in the two subgenomes. Out of the 6,484 ACRs near WGD genes in maize1, 2,742 (42.2%) overlap genes (gACRs, genic ACRs), 1,936 (29.8%) are within 2 kb of genes (pACRs, proximal ACRs), and 1,806 (27.8%) are >2 kb from a gene (dACRs, distal ACRs) (fig. 6*a*). Maize2 has similar proportions of gACRs, pACRs, and dACRs as does maize1 (fig. 6*a*). Given that biased fractionation is more pronounced in chromosomal arms (figs. 2 and 3), we compared ACRs near WGD genes between maize1 and maize2 in different genomic locations. We observed a significantly higher enrichment of gACRs, pACRs, and dACRs of maize1 only in chromosomal arms. No significant difference with respect to the enrichment of ACRs between maize1 and maize2 in pericentromeric regions (fig. 6*b*and*c*, and supplementary fig. S14, Supplementary Material online). Further comparison of the chromosome accessibility of these ACRs between chromosomal arms and pericentromeric regions indicate that maize1 ACRs are more accessible than maize2 but only in chromosomal arms (fig. 6*d*). Because *cis*-regulatory elements residing in ACRs regulate their target genes through chromatin loops (Li et al. 2019; Peng et al. 2019; Sun et al. 2020), we further compared the numbers of chromatin loops from the Hi-C-seq and HiChip-seq data (Ricci et al. 2019). Our data show that chromatin loops are enriched in chromosomal arms of maize1 relatively to maize2 arms, but no differences in the numbers of loops between maize1 and maize2 were detected in pericentromeric regions (supplementary fig. S15, Supplementary Material online).

In an attempt to shed light on the evolutionary forces that drive the difference of ACRs and duplicated genes, we further analyzed ACRs in maize that have syntenic ACRs in sorghum using publicly available data (Lu et al. 2019). We hypothesize that like maize1 gene coding sequences, maize1 ACRs have undergone a higher level of selective constraint. To test this, we classified 2,205 syntenic ACRs near WGD genes into (1) 381 paired ACRs that both copies have been retained, (2) 909 ACRs that are retained in maize1 and their homoeologous ACRs are lost in maize2, and (3) 534 ACRs that are retained in maize2 and their homoeologous ACRs are lost in maize1. In addition, we grouped the 1,350 syntenic ACRs near singleton genes into (4) 1,014 ACRs that are retained in maize1 and their homoeologous ACRs are lost in maize2, and (5) 336 ACRs that are retained in maize2 and their homoeologous ACRs are lost in maize1 (supplementary fig. S13, Supplementary Material online). We next calculated the frequencies of sequence substitution (K) of the 381 ACR pairs relative to their syntenic ACRs in sorghum, which served as an outgroup. Consistent with the evolutionary pattern of duplicated genes, maize1 ACRs exhibit a significantly lower value of K than do maize2 ACRs when both ACRs are in chromosomal arms. However, when both ACRs are in pericentromeric regions, no significant difference in K was observed between maize1 and maize2 (fig. 6*e*). These observations indicate that maize1 ACRs have been subject to a higher level of selective constraints than their maize2 homoeologous ACRs, but only when they are in chromosomal arms.

**Fig. 7.**
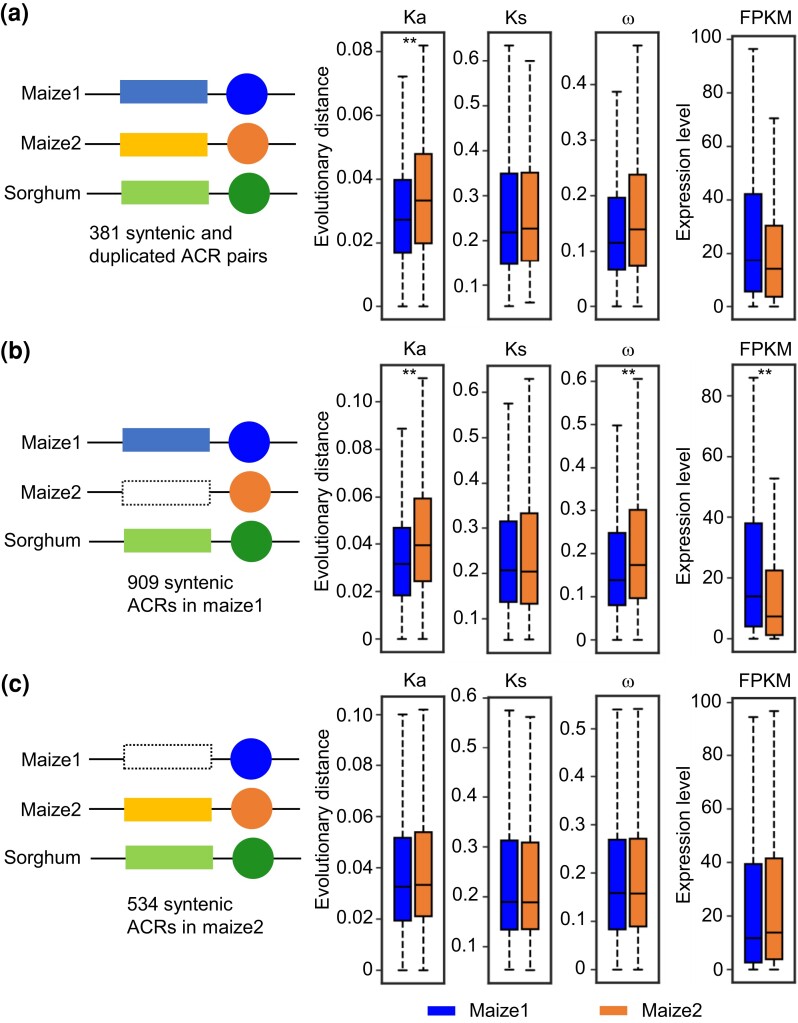
Presence or absence of accessible chromatin regions (ACRs) may have affected the biased evolution and expression of their flanking duplicated genes. (*a*) Both two duplicated ACRs retained in maize. (*b*) Maize1 ACRs retained and their maize2 homoeologous ACRs lost. (*c*) Maize1 ACRs lost and their maize2 homoeologous ACRs retained. Only syntenic ACRs between maize and sorghum near whole-genome duplicated genes were used here. Rectangles represent ACRs, and circles indicate duplicated gene pairs. Dotted rectangles indicate the ACRs are lost. The evolutionary distances (Ka, Ks, and ω) and expression values (FPKM, fragments per kilobase of exon per million mapped fragments) were compared between maize1 and maize2 homoeologous genes. The statistical analysis was conducted using Student’s paired *t*-test. **P < 0.01.

To further understand the interaction between ACRs and their target genes, we asked whether presence or absence of ACRs have impacted the evolution and expression of their target genes. We compared the values of Ka, Ks, ω, and FPKM of the maize1 and mazie2 duplicated gene pairs nearest to these syntenic ACRs. When the duplicated pairs of both ACRs and genes are retained (Category 1, fig. 7*a* and supplementary fig. S13, Supplementary Material online), Ka of maize1 genes is significantly lower than that of their maize2 homoeologs. In contrast, we find no significant difference in Ks, ω, or FPKM between this subset of maize homoeologs (fig. 7*a*), suggesting these pairs of genes have experienced similar levels of purifying selection. When maize2 ACRs are lost and only maize1 ACRs are retained (Category 2, fig. 7*b* and supplementary fig. S13, Supplementary Material online), both Ka and ω of maize1 genes are significantly lower than those of maize2 genes (fig. 7*b*), indicating that these maize1 genes have experienced an overall higher level of purifying selection than maize2 genes. Furthermore, these maize1 genes also exhibit a significantly higher level of expression than their maize2 homoeologs. In contrast, when maize1 ACRs are lost and only maize2 ACRs are retained (Category 3, fig. 7*c* and supplementary fig. S13, Supplementary Material online), no significant differences in Ka, Ks, ω, or FPKM were detected between the homoeologs (fig. 7*c*), indicating no biased evolution in these pairs of genes. This also suggests that these ACRs play a role in the dominance of these maize1 genes, and loss of these ACRs is associated with the relaxed selection of this subset of maize1 genes.

Finally, we compared chromosome accessibility, evolutionary distances (K, Ka, Ks, and ω), and expression values within maize1 subgenome (Categories 1, 2, and 4), and within maize2 subgenome (Categories 1, 3, and 5, supplementary fig. S13, Supplementary Material online). In both maize1 and maize2, paired syntenic ACRs have an average higher level of chromatin accessibility and a significantly lower level of sequence substitution K than singleton syntenic ACRs, which is consistent with the pattern observed between WGD genes and singleton genes (supplementary figs. S5 and S6, Supplementary Material online). In both maize1 and maize2, WGD genes have lower values of Ka and ω, and are expressed at significantly higher levels than are singleton genes. In contrast, no significant differences in Ks were detected between WGD genes and singleton genes in maize1, whereas the former exhibits a significantly higher value of Ks than the later in maize2 (supplementary fig. S16, Supplementary Material online). Overall, our data show that when the duplicated ACR pairs and gene pairs are both retained in the syntenic regions, both of members of these pairs of genes exhibit the slowest rate of evolutionary change, suggesting that they have functional correlation during the evolutionary history.

## Discussion

Our most striking observation is that we find no significant bias of most of the measured variables when comparing maize1 and maize2 homoeologs and their associated ACRs when maize1 genes were in pericentromeric regions (figs. 2*b*, 3*b*, 4*b*, 5*b*, and 6*e*). Maize1 WGD genes do not exhibit any evidence of stronger purifying selection than their maize2 homoeologs when both are in pericentromeric regions (fig. 2*b*). Pericentromeric regions are generally recombination suppressed, in which natural selection against deleterious mutations is poorly efficient (Gaut et al. 2007; Charlesworth 2012). Recombination can also cause point mutations (Lercher and Hurst 2002; Rousselle et al. 2019). Therefore, both Ka and Ks evolve more slowly in gene copies in recombination-suppressed pericentromeric regions than in homoeologous copies in chromosomal arms, as has been noted previously in soybean (Du et al. 2012). However, this is not what we observed here. When maize1 genes are in chromosomal arms and their maize2 homoeologs are in pericentromeric regions, we expected to see higher mutation rates for maize1. Instead, we detected a lower Ka and no significant difference in Ks for maize1 genes (fig. 2*c*), suggesting that the effect of purifying selection on the function of maize1 genes is stronger than that of recombination on the evolution of the homoeologs in maize. This result is consistent with a previously proposed model suggesting that biased fractionation is a result of purifying selection acting preferentially against deletion alleles of higher expressed duplicated copies (Schnable et al. 2011). Interestingly, we find that the expression dominance of maize1 over maize2 was only significant when maize1 genes are located in chromosomal arms, regardless of where their maize2 homoeologs are located (fig. 3*a*and*c*, left panels), suggesting chromatin environment has a significant effect on gene dominance. This may be due to differences in recombination. The major evolutionary advantage of recombination is thought to be that it breaks up associations between linked loci. Such linkage hinders the action of purifying selection and thus increases the fixation rate of deleterious mutations, resulting in increased ω (Webster and Hurst 2012; Bolivar et al. 2016). Because of lower rates of recombination in pericentromeric regions, deleterious mutations are less efficiently purged. Because of this, selection is expected to be weaker for maize1 genes in pericentromeric regions than in chromosomal arms. We hypothesize that because maize2 genes have already been subject to relaxed selection, accumulation of deleterious mutations in these genes may not have significant effects on them no matter where they are located. We suggest that maintenance of genome dominance requires that selection can effectively purge mildly deleterious alleles before they become fixed. In chromosome arms, this occurs preferentially in maize1 genes, resulting in a long-term maintenance of dominance. However, because of reduced recombination, dominance cannot be maintained in pericentromeric regions. To test this, we classified all duplicated genes into higher and lower recombination groups. Of the 4,578 duplicated pairs, 2,718 (59.4%) show recombination differences of at least two-fold and were dubbed higher and lower recombination genes. We found that on average, higher recombination genes were expressed at higher levels. Furthermore, TEs are farther from and less abundant near the higher recombination genes. However, such differences are not significant (supplementary fig. S17, Supplementary Material online) probably because the recombination rate data were generated from recently mapping populations (Liu et al. 2009), and the differentiations of these duplicated genes were the outcome of around 12 My evolution. In addition, we detected no significant differences with respect to GC content between maize1 and maize2 genes (supplementary fig. S18, Supplementary Material online).

Transposable element insertions are likely to be one of the deleterious mutations that contribute to genome dominance because silenced TEs often have deleterious effects on expression of their neighboring genes (Hollister and Gaut 2009). It has been hypothesized that the two subgenomes prior to the allotetraploidization event in maize were distinct with respect to TE distribution such that one subgenome had lower overall levels of expression due to a greater density of silenced TEs near genes (Woodhouse et al. 2014; Zhao et al. 2017). In cases in which the genes were functionally redundant, it is hypothesized that the member of a pair with a lower level of expression would be more likely to be lost. Due to dosage constraints, some classes of genes retain both copies (Freeling and Thomas 2006; Thomas et al. 2006). In these cases, purifying selection on the gene with the lower expression level, either due to TE insertion or any other deleterious mutations, was lower, resulting in relaxed selection against additional deleterious mutations or subsequent TE insertions (Freeling and Thomas 2006; Thomas et al. 2006; Wendel et al. 2018). Given that silenced TEs near genes have deleterious effects on neighboring gene expression (Hollister and Gaut 2009), purifying selection is thought to purge TE insertions from gene-rich chromosomal regions, which led to the accumulation of TEs in low-recombining pericentromeric regions (Wright et al. 2003). It is also possible that the accumulation of at least some of these TEs is caused by biased insertion of these TE sequences in these recombination-suppressed regions (Wright et al. 2003; Tian et al. 2012). However, what is more relevant here is our observation that TEs are closer to genes in these regions (fig. 4), and TEs have accumulated at similar abundances and at similar distances around homoeologous genes in pericentromeric regions in the two subgenomes (fig. 4). If we assume that they have similar effects on the expression of the genes, this is consistent with the idea that within pericentromeres, both copies of duplicated genes in these regions have experienced similar levels of selective constraint. This is also reflected in differences in abundance of 24 nt small RNAs and CHH methylation in the same regions of the two subgenomes (fig. 5).

It should be emphasized that the current regions classified as pericentromeric regions or chromosomal arms may not be the same regions in the two progenitor genomes given that large numbers of rearrangements have occurred in maize after the tetraploid event. These rearrangements have led to transitions of many genomic regions from chromosomal arms to pericentromeric regions or vice versa (Swigonova et al. 2004; Wei et al. 2007; Wang and Bennetzen 2012). It is also possible that the two homoeologs both currently located in pericentromeric regions were less distinct prior to polyplodization because they were both located in heterochromatin when polyploidy occurred and were initially expressed at the similar level because both members of each gene pair were similarly compromised and therefore had an equal chance of being lost and an equal chance of experiencing a relative reduction in purifying selection. Finally, it remains a possibility is that since selection is weaker in pericentromeric regions, bias may also occur in these regions its effects would take longer to manifest themselves.

Another interesting observation is the coevolution of ACRs and their flanking genes. It is not surprising that these ACRs are largely enriched in euchromatic chromosomal arms, which contain many active genes. However, even correcting for gene density, ACRs are more numerous in euchromatin, and, like the genes they are associated with, ACRs in maize1 differ with respect to number, level of chromatin accessibility, and evolutionary distance (K) to those in maize2. In contrast, none of these differences between subgenomes are observed in pericentromeric regions (fig. 7). Our data also indicate that loss of maize2 ACRs may not have had a large effect on the evolution and expression of their flanking genes, whereas loss of maize1 ACRs is associated with a reduction in purifying selection and expression of maize1 genes relative to their maize2 homoeologs (fig. 7*b*and*c*), suggesting that these ACRs near maize1 genes are important to maintain the gene dominance. These data would also be indicative of coevolution between regulatory elements and their target genes that is supportive of the Gene Balance Hypothesis. This hypothesis states that imbalance in macromolecular complexes and in signaling networks will affect the function of the whole and lead to fitness defects (Veitia et al. 2008; Birchler and Veitia 2010, 2012). Because ACRs harbor *cis*-regulatory elements that control the expression of genes, ACRs and their target genes are in balance with each other. Natural selection purges changes in either one because changes in either will result in reduced function. Given that maize1 genes contribute more to phenotypic traits, they have been retained more frequently and have undergone a stronger purifying selection (Pophaly and Tellier 2015; Zhao et al. 2017), their regulatory elements are also more retained and more conserved (figs. 6 and 7). Although the causes and consequences of subgenome differentiation in maize remain to be more fully elucidated, our study points out that chromatin environment, TEs and their associated marks, and regulatory elements are all important determinants shaping the patterns of divergence of homoeologous genes retained in the two subgenomes.

## Materials and Methods

### Separation of Chromosomal Arms and Pericentromeric Regions of Maize Chromosomes

The rough positions of chromosomal arms and pericentromeric regions of the ten maize chromosomes were defined based on the gene and TE densities as well as recombination rates following our previous method (fig. 1; Zhao et al. 2021). Based on the annotation of genes and TEs, gene densities (number of genes/Mb) and repeat length (Mb/Mb) were measured in 1 Mb windows with 500 kb shifts along each chromosome. Recombination rates (cM/Mb) were determined based on 6,257 genetic markers in the integrated map previously described (Liu et al. 2009). Because recombination is generally suppressed, gene density is lower, and TE density is higher in pericentromeric regions, we manually separated each chromosome into two arms and one pericentromeric region (fig. 1). Presumed centromeric positions were determined by the functional centromere positions previously mapped (Wolfgruber et al. 2009). The sequences of the functional centromeres were extracted from the B73 v1 genome, and mapped to the B73 v4 reference genome using BLAST (Altschul et al. 1997).

### Identification of Homoeologous and Syntenic Genes and ACRs

The syntenic gene list of maize (v4) and sorghum were obtained from previously published data (Zhang et al. 2017). We only kept the maize genes with syntenic relationships in sorghum. To make the data more accurate, genes involved in tandem duplication were first removed because of their ambiguous duplication status. In addition, we filtered the genes with the values of Ks <0.05 and the ratio (ω) of Ka to Ks >2.0 between maize and sorghum. The final data set in this study includes a total of 4,578 duplicated gene pairs and 11,554 singleton genes (supplementary fig. S1 and table S1, Supplementary Material online). These 4,578 duplicated gene pairs were further classified into four categories based on their genomic locations in chromosomal arms and pericentromeric regions: M1-arm versus M2-arm, both of the two homoeologous genes in chromosomal arms, M1-peri versus M2-peri, both of the two homoeologous genes in pericentromeric regions, M1-arm versus M2-peri, maize1 genes in chromosomal arms and maize2 genes in pericentromeric regions, and M1-peri versus M2-arm, maize1 genes in pericentromeric regions and maize2 genes in chromosomal arms. To compare the characters between subgenomes, the maize v4 genomes were split into blocks. Small blocks with fewer than five genes were excluded from further analysis. This left a final set of 119 duplicated block pairs.

The maize and sorghum ACRs were downloaded from the published studies (Lu et al. 2019; Ricci et al. 2019). To determine duplicated ACRs between subgenomes and syntenic ACRs between maize and sorghum, we compared the sequence similarities of ACRs within maize, and between maize and sorghum using BLAST (Altschul et al. 1997). ACRs with sequence similarities in the duplicated regions within maize or in the syntenic regions between maize and sorghum were defined as duplicated ACRs or syntenic ACRs.

### Estimation of Evolutionary Distance and GERP Scores

We followed the pipelines previously described to analyze sequence divergence of syntenic genes and ACRs between maize and sorghum (Zhao et al. 2015, 2017, 2018). Homologous nucleotide sequences were aligned using MUSCLE or ClustalW by default parameters (Thompson et al. 1994; Edgar 2004). The unexpected stop codons generated by the alignment process but not present in the original sequence were replaced with “–” as ambiguous nucleotides before the estimation of sequence divergence. Ka and Ks of homologous genes and nucleotide sequence divergence K of ACRs were estimated with the yn00 and baseml modules in the PAML program (Yang 2007).

We calculated GERP scores for each site across the B73 reference genome and determined the deleterious alleles as alleles different from the ancestor alleles in the *S. bicolor* genome following previous studies (Rodgers-Melnick et al. 2015; Wang et al. 2017; Yang et al. 2017). Only nonsynonymous SNP sites with GERP scores >0 were retained as putatively deleterious loci. To mitigate reference bias, we calculated the average value of the genetic load for these putatively deleterious loci in the 4,578 duplicated gene pairs (9,156 duplicated genes) across the maize diversity panel (Bukowski et al. 2018). The genetic load was measured as the average number of deleterious alleles divided by the total length of the gene body.

### Analysis of Transcription and Protein Abundance of Homoeologous Genes

The raw data from 24 maize tissues were downloaded from public data sets (Sekhon et al. 2013; Eveland et al. 2014). The reads were aligned to the maize reference genome (v4) using HISAT version 2.1.0 (Kim et al. 2019). Only unique reads were kept to measure gene expression values, which were generated using cufflinks v2.2.1 (Trapnell et al. 2012). The horse race method was used to measure the expression dominance of the two duplicated genes of a pair in different chromatin environments across these 24 tissues (Schnable et al. 2011; Woodhouse et al. 2014; Zhao et al. 2017). In each tissue, we compared the number of duplicated gene pairs in which either maize1 or maize2 dominated expression following the method described in previous studies (Schnable et al. 2011; Woodhouse et al. 2014; Zhao et al. 2017). Dominant expression was defined as instances in which expression of one homoeolog is two-fold or greater than the expression of the other homoeolog in that tissue. For example, in each tissue, for a given duplicated pair, if the expression value of the maize1 gene is two-fold higher than its counterpart maize2 gene, we considered the maize1 gene won the horse and dominated expression, or vice versa.

The data set of protein abundance from 148 samples of 23 tissues was obtained from public papers (Walley et al. 2016; Walsh et al. 2020). Gene IDs were converted from version 2 to version 4 of the B73 genome. The horse race method with the same two-fold cutoff was used here to determine protein dominance in each sample.

### Analysis of TEs, 24 nt Small RNAs and DNA Methylation

A total of 1,526 consensus and exemplar TEs from the maize TE consortium were obtained from previous published manuscripts (Baucom et al. 2009; Schnable et al. 2009). These TE exemplars were used to search against the maize v4 genome using RepeatMasker with a divergence value of <20%. The distance of TEs overlapping with genes was considered as 0. The TE proportion was measured in each 100 bp window with 10 bp increments in the 2 kb upstream and downstream regions of duplicated genes.

The raw reads of small RNA data from four maize tissues (tassel, ear, seedling, and root) obtained from previous research were first mapped to the Rfam database (v14.6) to remove rRNAs, tRNAs, snRNAs, and snoRNAs (Bousios et al. 2016). After filtration, the remaining reads were mapped to the maize reference genome (v4) using Bowtie only allowing unique and perfect matches (Langmead et al. 2009). The values of small RNAs were normalized to transcripts per 10 million uniquely and perfectly mapped reads (TP10M). Twenty-four nucleotide small RNAs were measured in a 100 bp sliding window moving in 10 bp increments through the 2 kb upstream and downstream regions of the duplicated genes in maize1 and maize2 following the methods previously described (Woodhouse et al. 2014; Zhao et al. 2017).

Clean reads of whole-genome bisulfite sequencing data from four maize tissues (ear shoot, shoot apex, anther, and third seedling leaf) were aligned against the maize B73 v4 genome using Bismark under following parameters (-n 2, -I 50, -N 1; Krueger and Andrews 2011; Eichten et al. 2013; Li et al. 2015). Polymerase chain reaction duplicates were removed using Picardtools. Additional packages including Bismark methylation extractor, bismark2bedGraph, and coverage2cytosine under Bismark were used to extract the methylated cytosines, and to count methylated and unmethylated reads. The proportion of each type of methylation (CG, CHG, and CHH) was determined as weighted methylation levels in 50 bp windows without shifts of the 2 kb upstream and downstream regions of the duplicated genes (Krueger and Andrews 2011; Liu et al. 2021). Gene body methylation was measured on 40 equally sized bins, with bin size differing from gene to gene because of the different lengths.

### Analysis of Various Histone Modifications and Chromatin Loops

The mapping bed files of ChIP-seq data of H3K4me1, H3K4me3, H3K27me3, H3K36me3, H3K9ac, H3K27ac, H3K56ac, H3K9me2, and the histone variant H2A.Z were downloaded from previous research (Ricci et al. 2019; Long et al. 2021). The signals (number of reads) of each histone were measured in a 100 bp window of the 2 kb upstream and downstream regions and gene bodies of the duplicated genes following the formula log_2_(treat reads + 1)/(input reads + 1).

Chromatin loops connecting distal ACRs and their target genes in the leaf tissue were downloaded from a published paper (Ricci et al. 2019). These loops were obtained from three different types of Hi-C data (Hi-C-seq, H3K4me3-HiChIP-seq, and H3K27me3-HiChIP-seq). These chromatin loops were classified into chromosomal arms and pericentromeric regions of maize1 and maize2 based on their physical locations on each chromosome.

## Supplementary Material

msac198_Supplementary_Data

## Data Availability

This project analyzed only data that have been previously reported in other publications (see Materials and Methods for public data references).
