# Supplementary material for: The Heterogeneity in the Landscape of Gene Dominance in Maize is Accompanied by Unique Chromatin Environments
Source: Mol Biol Evol. 2022 Sep 21;39(10):msac198. doi: 10.1093/molbev/msac198 (PMC9547528; doi:10.1093/molbev/msac198)
Supplement: msac198_Supplementary_Data [file msac198_supplementary_data.zip › Supplementary_Figures.pdf]

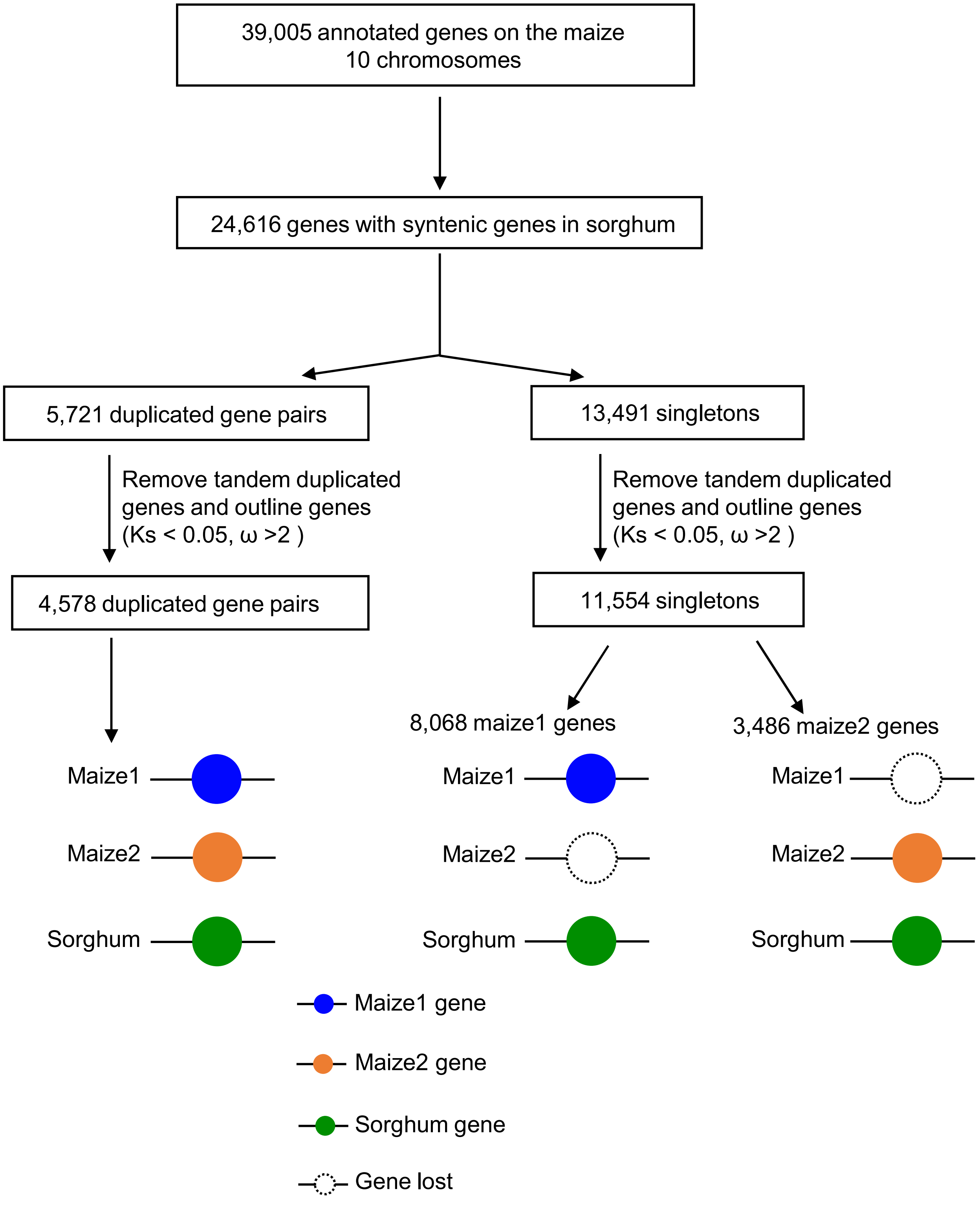

supplementary fig. S1. Bioinformatics pipeline to analyze WGD genes and singletons.

The whole pan-grass syntenic gene data set was downloaded from Zhang et al. 2017. WGD, whole genome duplication.

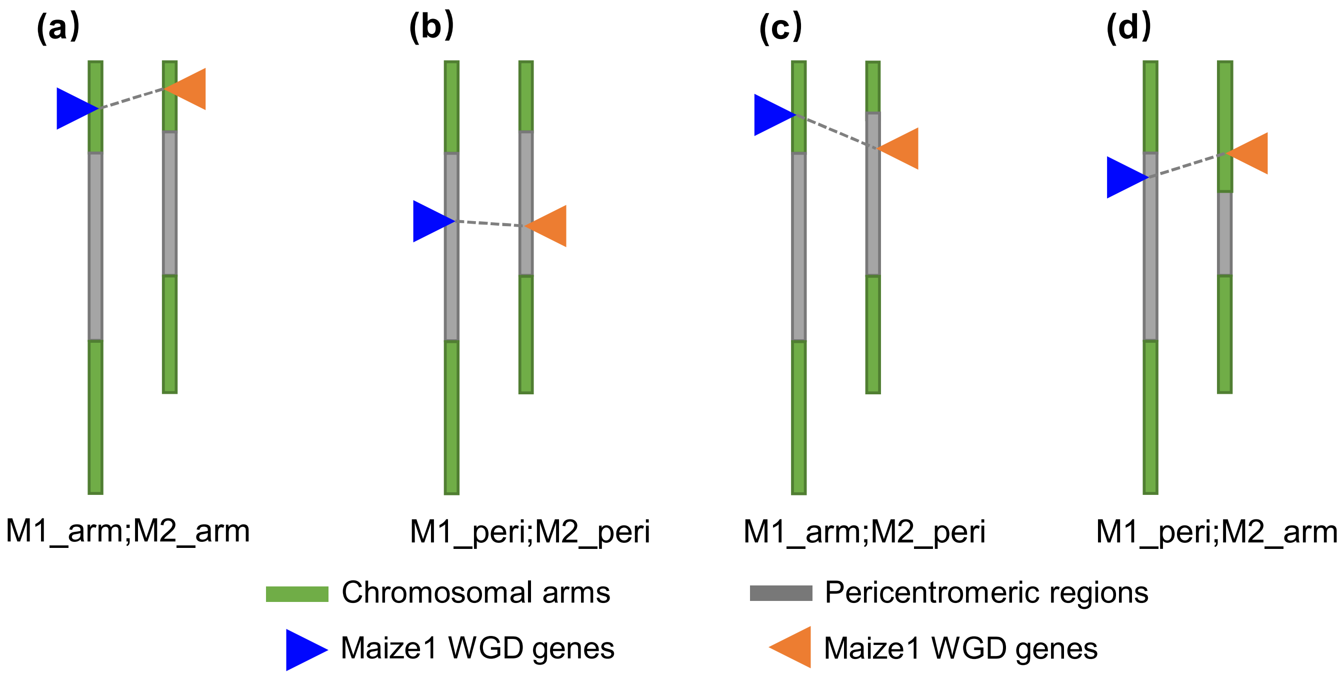

supplementary fig. S2. Schematic diagram showing the locations of duplicated gene pairs in the four categories.

(a) Both homoeologous genes in chromosome arms (M1_arm;M2_arm: 2,877 gene pairs).

(b) Both homoeologous genes in pericentromeric regions (M1_peri;M2_peri: 429 gene pairs).

(c) Maize1 genes in chromosome arms, and maize2 genes in pericentromeric regions (M1_arm;M2_peri: 720 gene pairs).

(d) Maize1 genes in pericentromeric regions, and maize2 genes in chromosome arms (M1_peri;M2_arm: 552 gene pairs).

WGD, whole genome duplication.

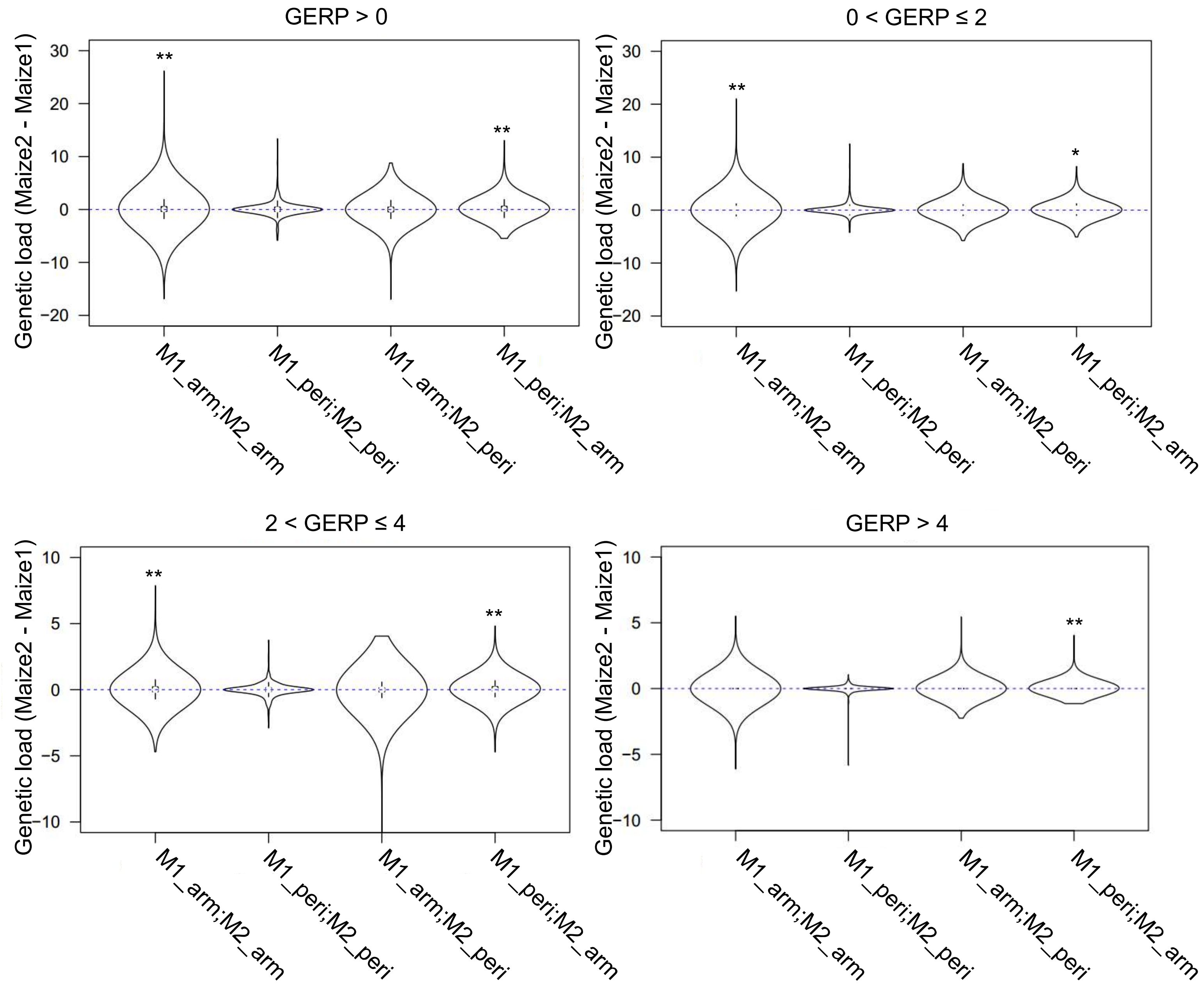

supplementary fig. S3. No significant difference with respect to the genetic load (deleterious alleles) between maize1 and maize2 genes in pericentromeric regions.

Nonsynonymous single nucleotide polymorphism (SNP) sites with genomic evolutionary rate profiling (GERP) score great than zero were determined as deleterious alleles. The genetic load was measured as the average number of deleterious alleles divided by the total length of the gene body. The statistical analysis was conducted using Student's paired *t* test. **, $P < 0.01$; *, $P < 0.05$.

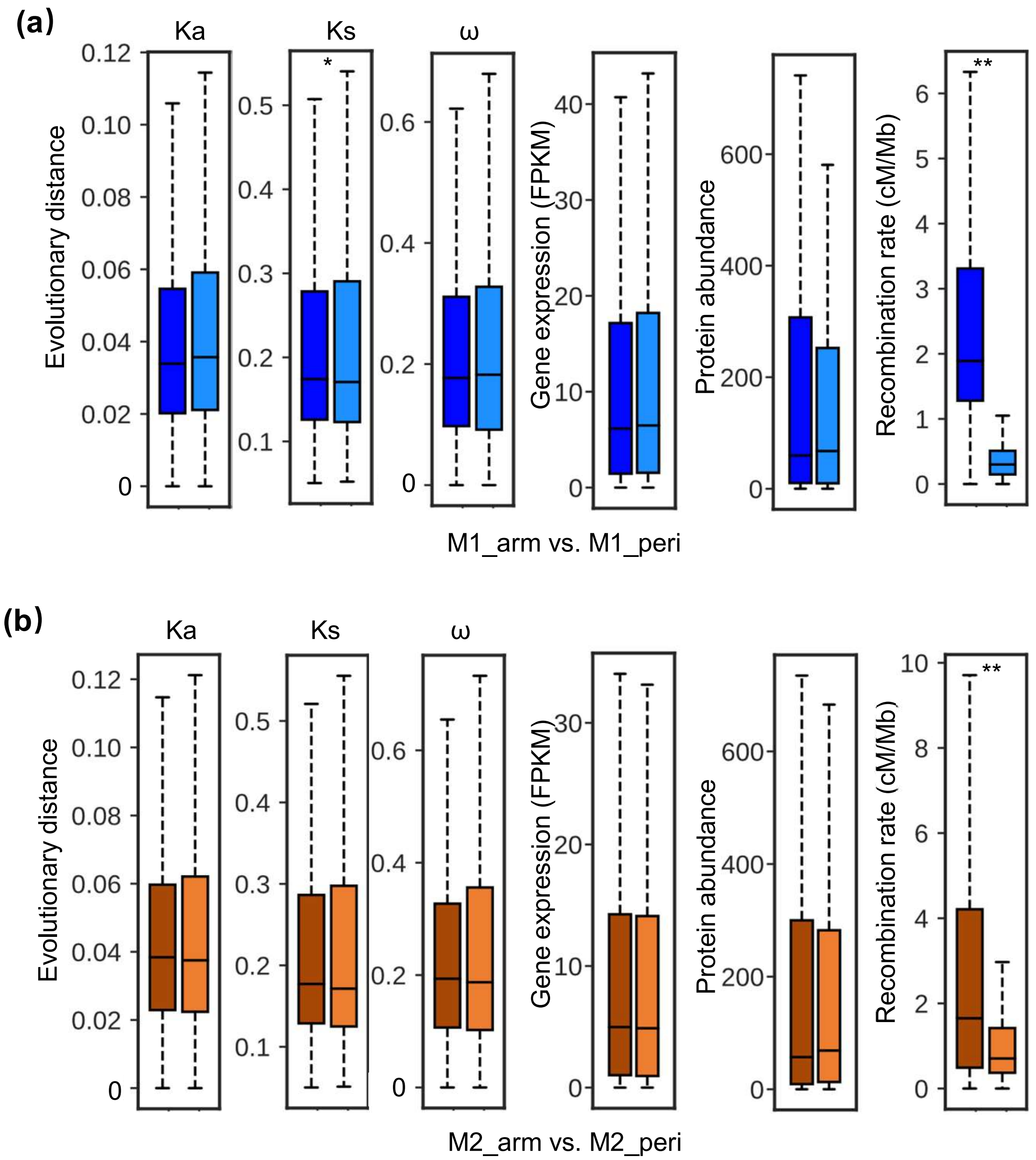

supplementary fig. S4. Comparisons of evolutionary distances and recombination rates between WGD genes in pericentromeric regions than in chromosomal arms.
(a) WGD genes in maize1 (M1_arm vs. M1_peri).
(b) WGD genes in maize2 (M2_arm vs. M2_peri).

The statistical analysis was conducted using Student's *t* test. **, $P < 0.01$; *, $P < 0.05$. WGD, whole genome duplication.

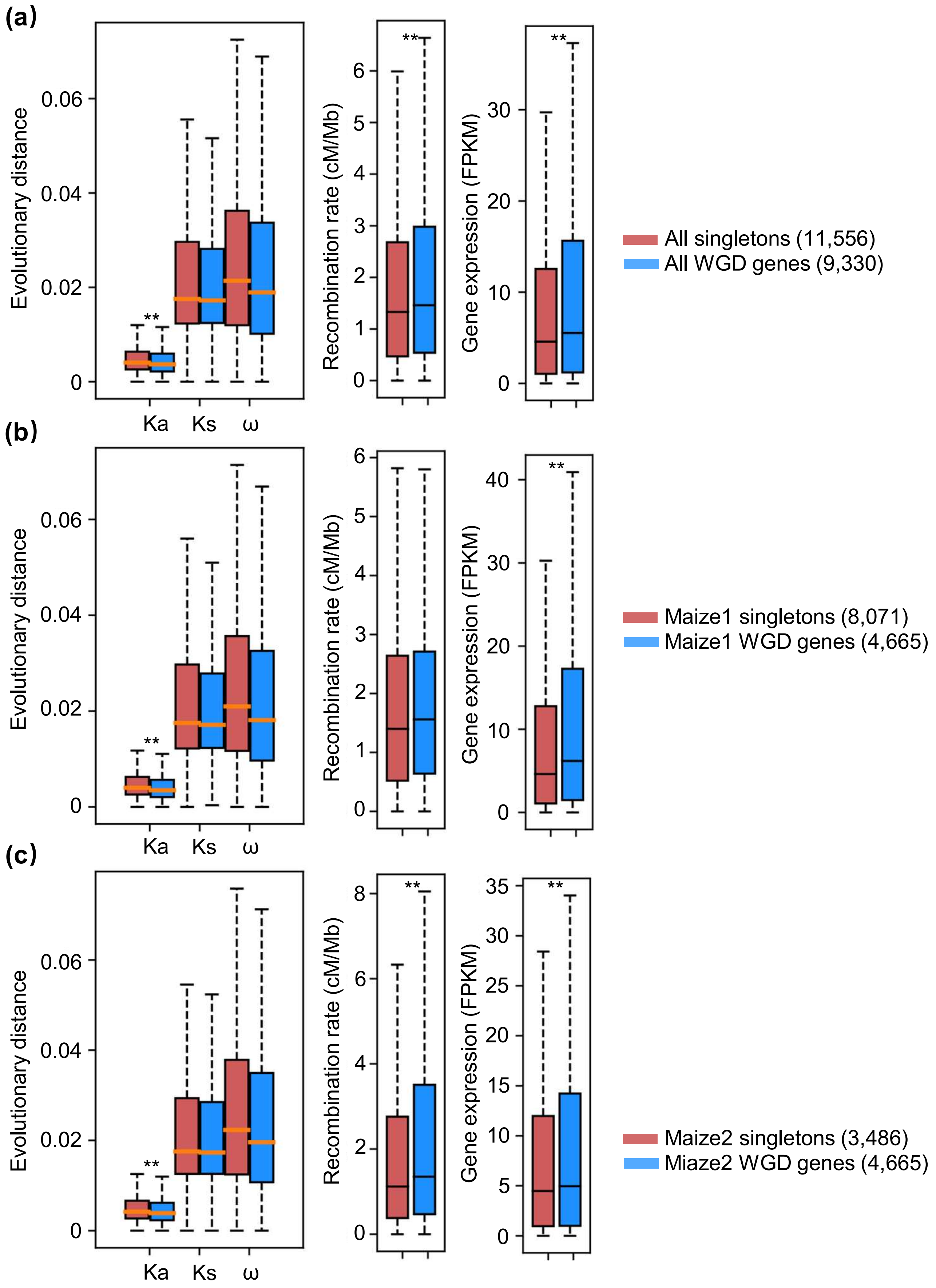

supplementary fig. S5. WGD genes exhibit significantly lower levels of nonsynonymous substitution (Ka) and significantly higher values of gene expression than do singletons.

(a) All singletons versus WGD genes in the maize genome.
(b) Singletons versus WGD genes in maize1.
(c) Singletons versus WGD genes in maize2.

The statistical analysis was conducted using Student's *t* test. **, $P < 0.01$; *, $P < 0.05$. WGD, whole genome duplication.

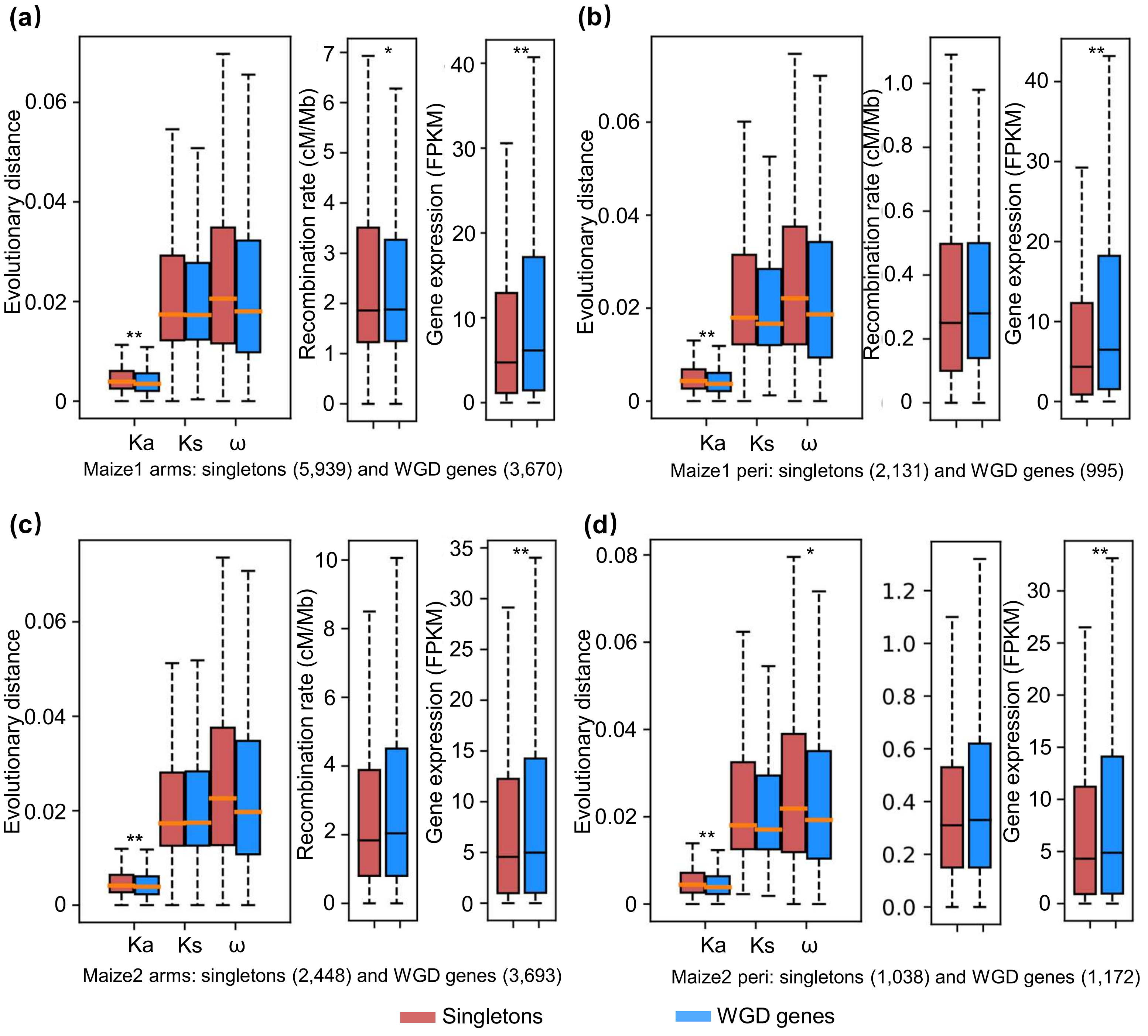

supplementary fig. S6. WGD genes exhibit significantly lower levels of nonsynonymous substitution (Ka) and significantly higher values of gene expression than do singletons in both chromosomal arms and pericentromeric regions.

(a) Singletons (5,939) versus WGD genes (3,670) in maize1 in chromosomal arms.
(b) Singletons (2,131) versus WGD genes (995) in maize1 in pericentromeric regions.
(c) Singletons (2,448) versus WGD genes (3,693) in maize2 in chromosomal arms.
(d) Singletons (1,038) versus WGD genes (1,172) in maize2 in pericentromeric regions.

The statistical analysis was conducted using Student's *t* test. **, $P < 0.01$; *, $P < 0.05$. WGD, whole genome duplication.

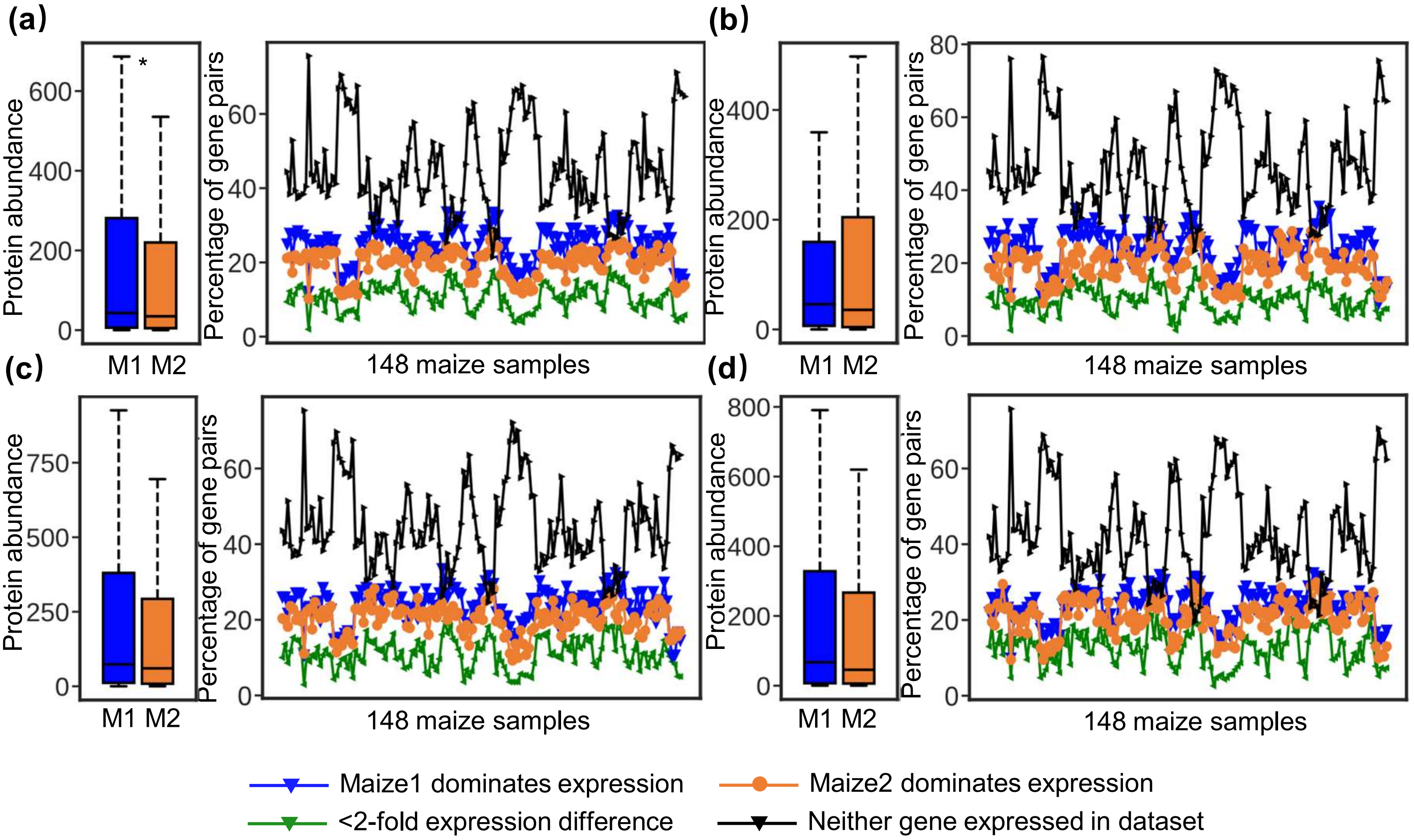

supplementary fig. S7. Biased protein abundance of maize1 in chromosomal arms.
(a) Both homoeologous genes in chromosome arms (M1_arm;M2_arm: 1,215 gene pairs).
(b) Both homoeologous genes in pericentromeric regions (M1_peri;M2_peri: 188 gene pairs).
(c) Maize1 genes in chromosome arms, and maize2 genes in pericentromeric regions (M1_arm;M2_peri: 280 gene pairs).
(d) Maize1 genes in pericentromeric regions, and maize2 genes in chromosome arms (M1_peri;M2_arm: 231 gene pairs).

Protein abundance data were obtained from previous research (Walley et al. 2016; Walsh et al. 2020) and is compared following the horse race method used for RNA expression. Only 1,914 duplicated gene pairs were used here because of the availability of the data. The statistical analysis was conducted using Student's paired *t* test. *, $P < 0.05$.

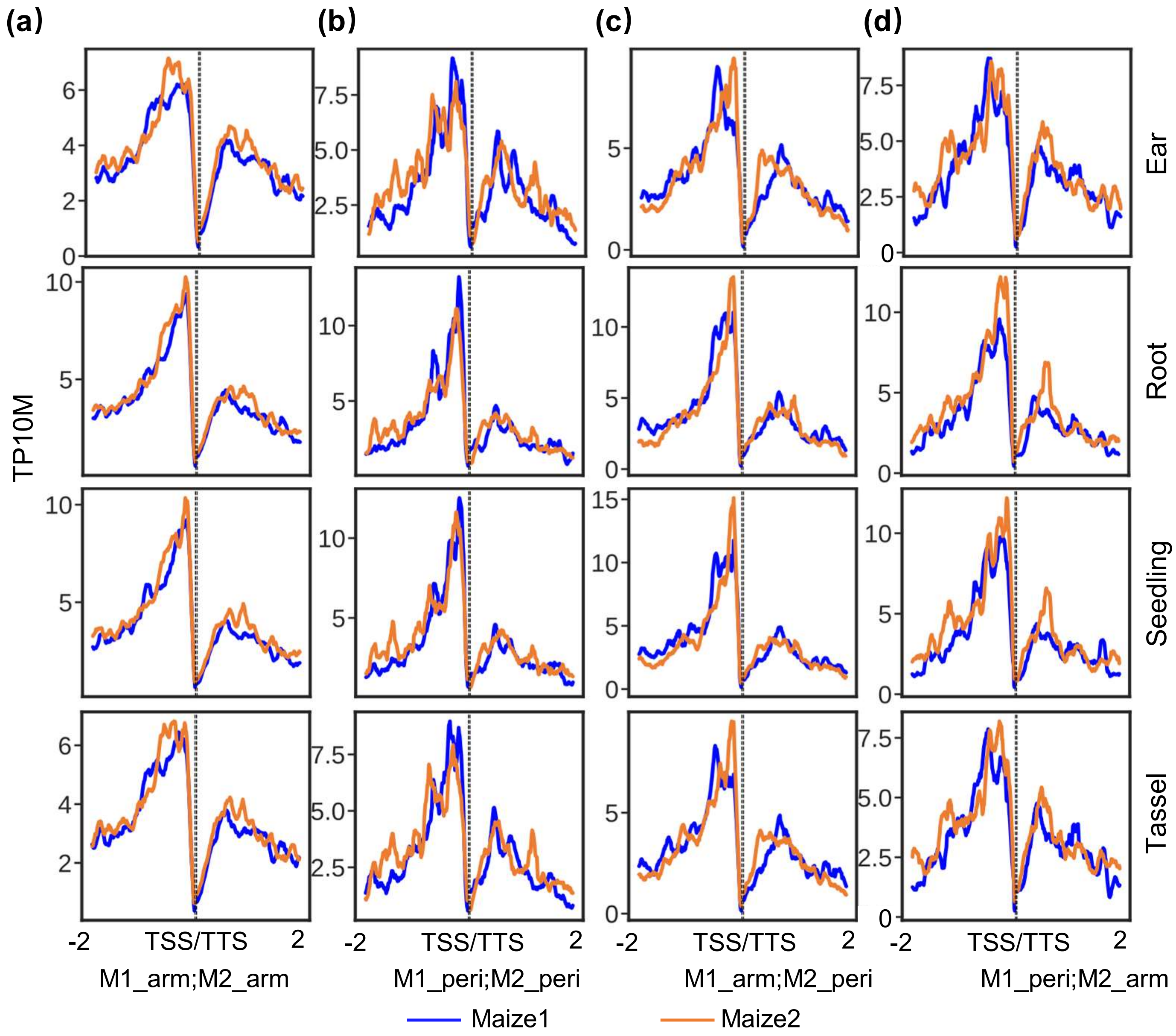

supplementary fig. S8. Distribution of 24 nucleotide small RNAs in different chromatin environments from four maize tissues.
(a) Both homoeologous genes in chromosome arms (M1_arm;M2_arm: 2,877 gene pairs).
(b) Both homoeologous genes in pericentromeric regions (M1_peri;M2_peri: 429 gene pairs).
(c) Maize1 genes in chromosome arms, and maize2 genes in pericentromeric regions (M1_arm;M2_peri: 720 gene pairs).
(d) Maize1 genes in pericentromeric regions, and maize2 genes in chromosome arms (M1_peri;M2_arm: 552 gene pairs).

Only uniquely and perfectly mapped 24 nucleotide small RNAs were averaged in a 100 bp sliding window moving in 10 bp increments of the 2 kb upstream and downstream regions of WGD genes following the methods previously described (Woodhouse et al. 2014; Zhao et al. 2017). TSS, transcription start site; TTS, transcription termination site. WGD, whole genome duplication.

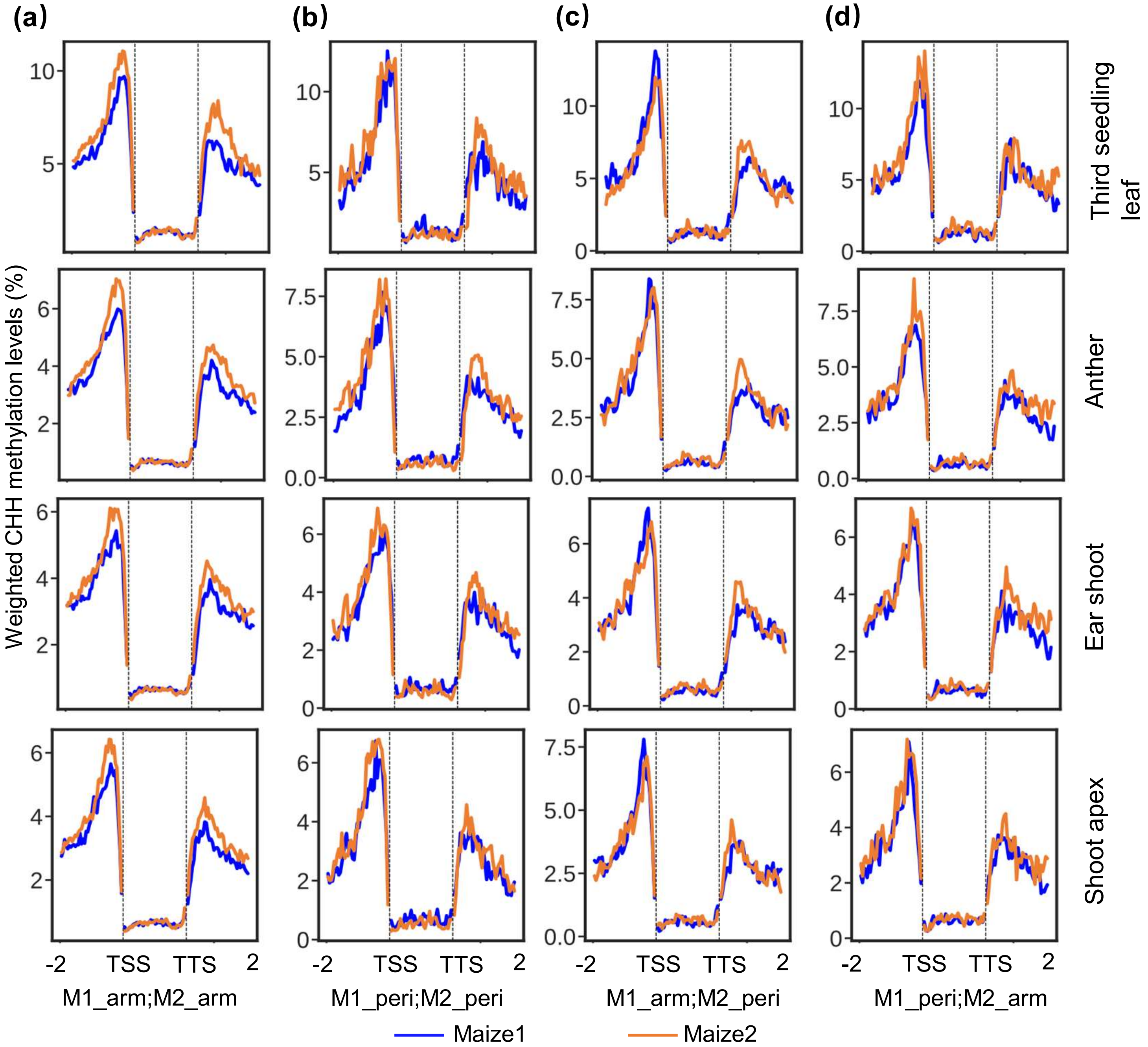

supplementary fig. S9. CHH methylation around and on duplicated gene pairs in different chromatin environments from four maize tissues.

(a) Both homoeologous genes in chromosome arms (M1_arm;M2_arm: 2,877 gene pairs).

(b) Both homoeologous genes in pericentromeric regions (M1_peri;M2_peri: 429 gene pairs).

(c) Maize1 genes in chromosome arms, and maize2 genes in pericentromeric regions (M1_arm;M2_peri: 720 gene pairs).

(d) Maize1 genes in pericentromeric regions, and maize2 genes in chromosome arms (M1_peri;M2_arm: 552 gene pairs).

Weighted methylation levels were calculated in a 50 bp window following the methods previously described (Schultz et al. 2012; Zhao et al. 2017). Each gene sequence was divided into 40 equally sized bins to measure the gene body methylation. Bin sizes differ from gene to gene because of the different lengths of genes. In total, four maize tissues (Eichten et al. 2013) were used here. TSS, transcription start site; TTS, transcription termination site.

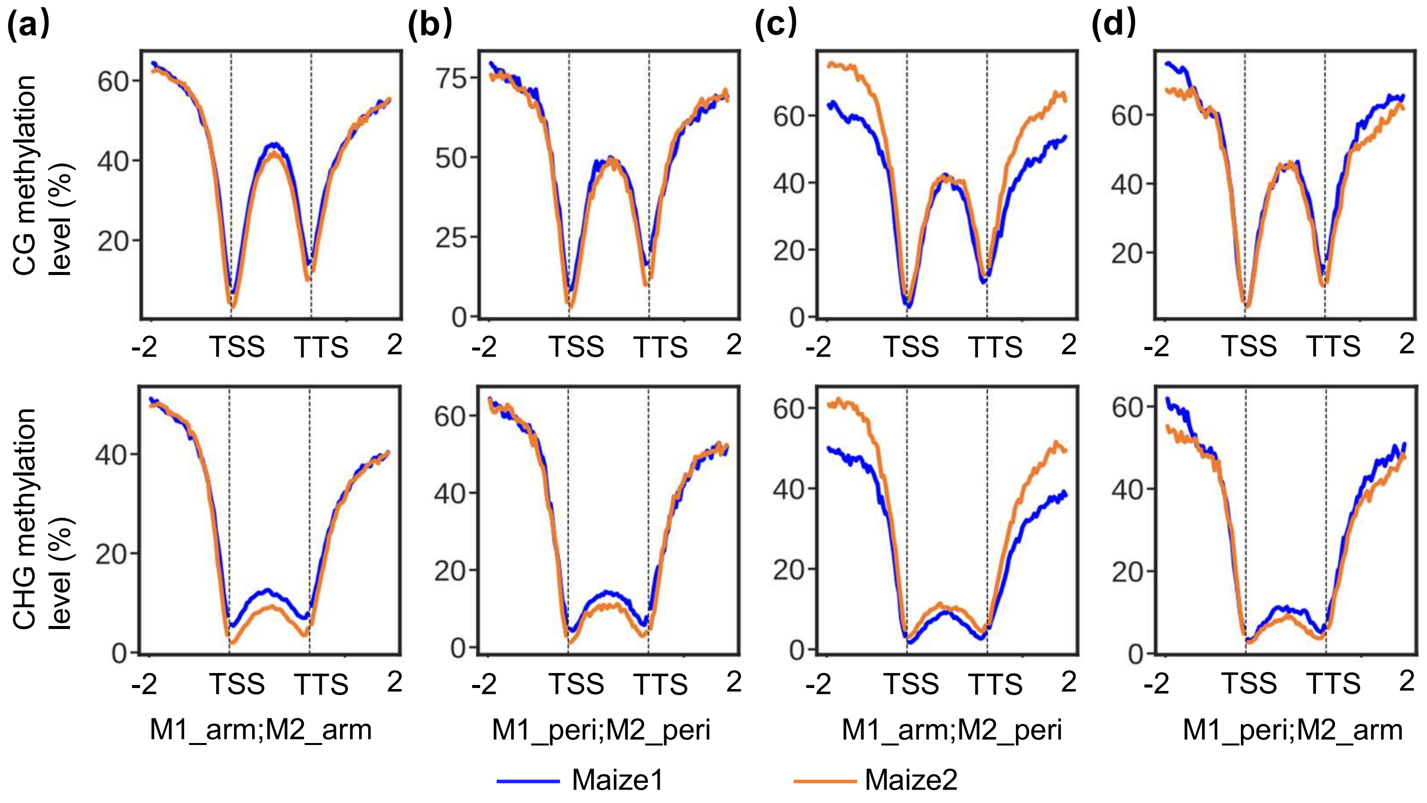

supplementary fig. S10. CG and CHG (H = A, T, or C) methylation levels between maize1 and maize2 homoeologous genes in different chromatin environments.
(a) Both homoeologous genes in chromosome arms (M1_arm;M2_arm: 2,877 gene pairs).
(b) Both homoeologous genes in pericentromeric regions (M1_peri;M2_peri: 429 gene pairs).
(c) Maize1 genes in chromosome arms, and maize2 genes in pericentromeric regions (M1_arm;M2_peri: 720 gene pairs).
(d) Maize1 genes in pericentromeric regions, and maize2 genes in chromosome arms (M1_peri;M2_arm: 552 gene pairs).

Weighted methylation levels were calculated in a 50 bp window following the methods previously described (Schultz et al. 2012; Zhao et al. 2017). Each gene sequence was divided into 40 equally sized bins to measure the gene body methylation. Bin sizes differ from gene to gene because of the different lengths of genes. In total, four maize tissues (Eichten et al. 2013) were used here. TSS, transcription start site; TTS, transcription termination site.

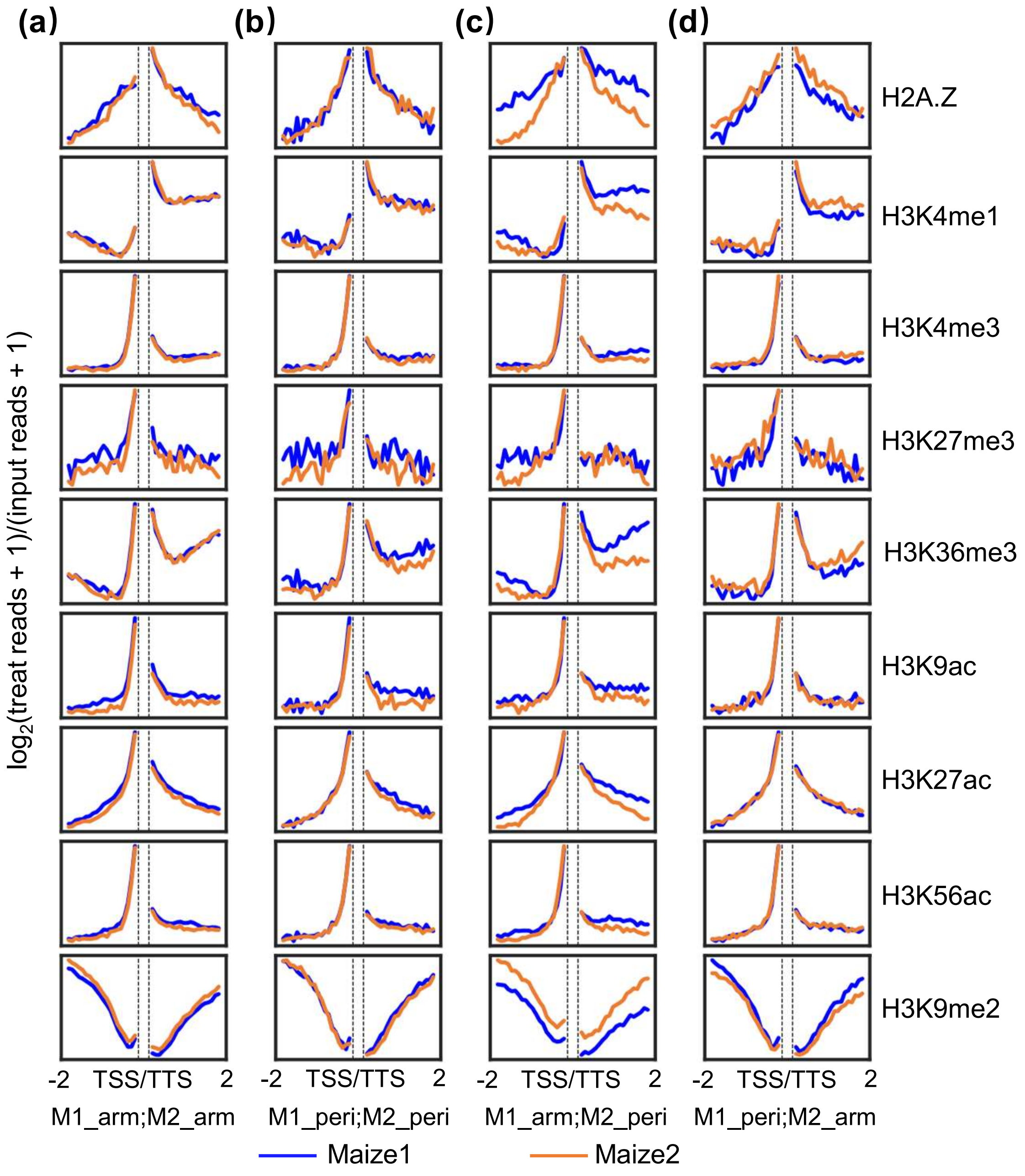

supplementary fig. S11. Distribution and abundances of various histone modifications around homoeologous genes in different chromatin environments.

(a) Both homoeologous genes in chromosome arms (M1_arm;M2_arm: 2,877 gene pairs).

(b) Both homoeologous genes in pericentromeric regions (M1_peri;M2_peri: 429 gene pairs).

(c) Maize1 genes in chromosome arms, and maize2 genes in pericentromeric regions (M1_arm;M2_peri: 720 gene pairs).

(d) Maize1 genes in pericentromeric regions, and maize2 genes in chromosome arms (M1_peri;M2_arm: 552 gene pairs).

TSS, transcription start site; TTS, transcription termination site.

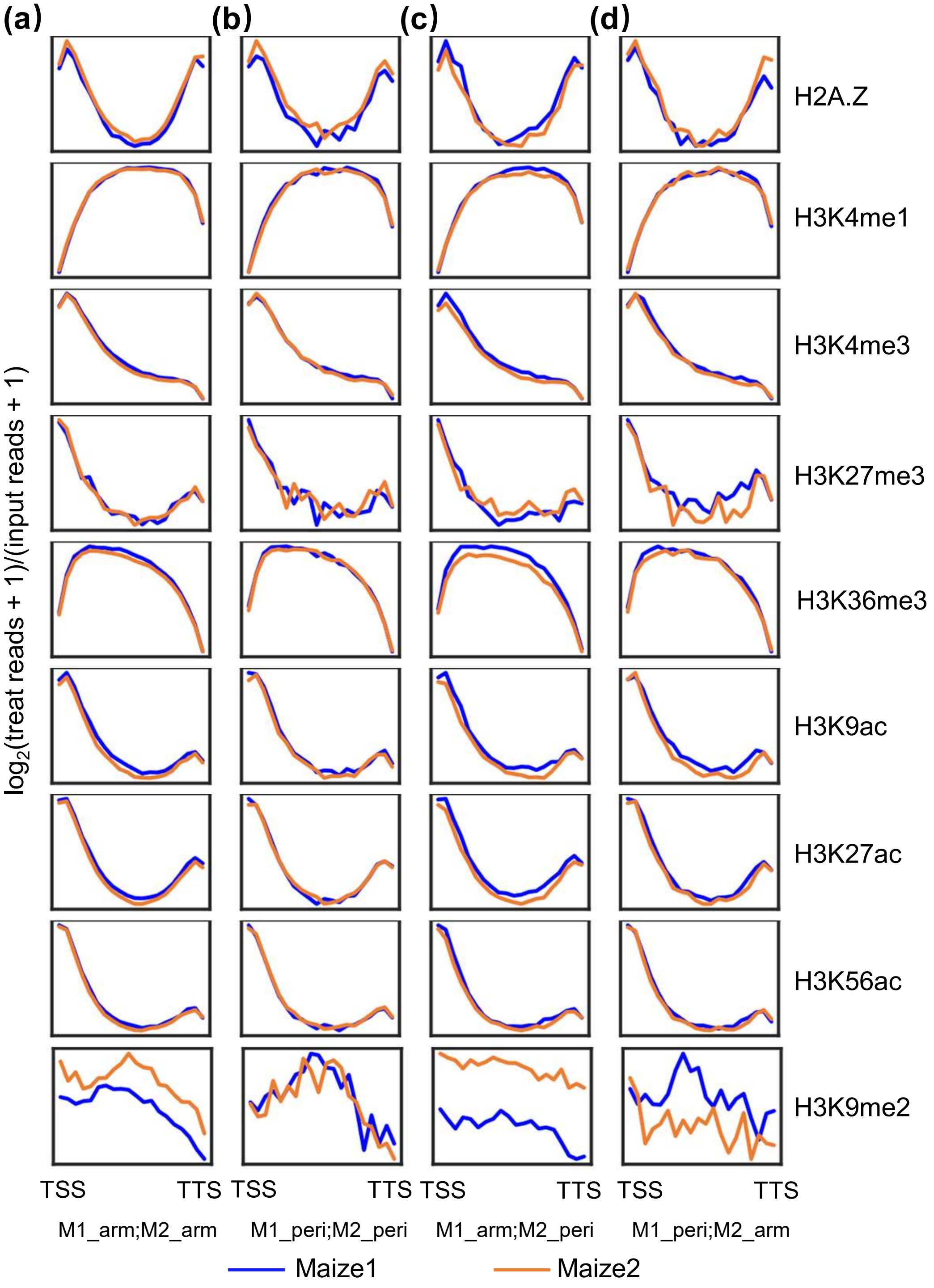

supplementary fig. S12. Distribution of various histone modifications on gene bodies in different chromatin environments.
(a) Both homoeologous genes in chromosome arms (M1_arm;M2_arm: 2,877 gene pairs).
(b) Both homoeologous genes in pericentromeric regions (M1_peri;M2_peri: 429 gene pairs).
(c) Maize1 genes in chromosome arms, and maize2 genes in pericentromeric regions (M1_arm;M2_peri: 720 gene pairs).
(d) Maize1 genes in pericentromeric regions, and maize2 genes in chromosome arms (M1_peri;M2_arm: 552 gene pairs).

TSS, transcription start site; TTS, transcription termination site.

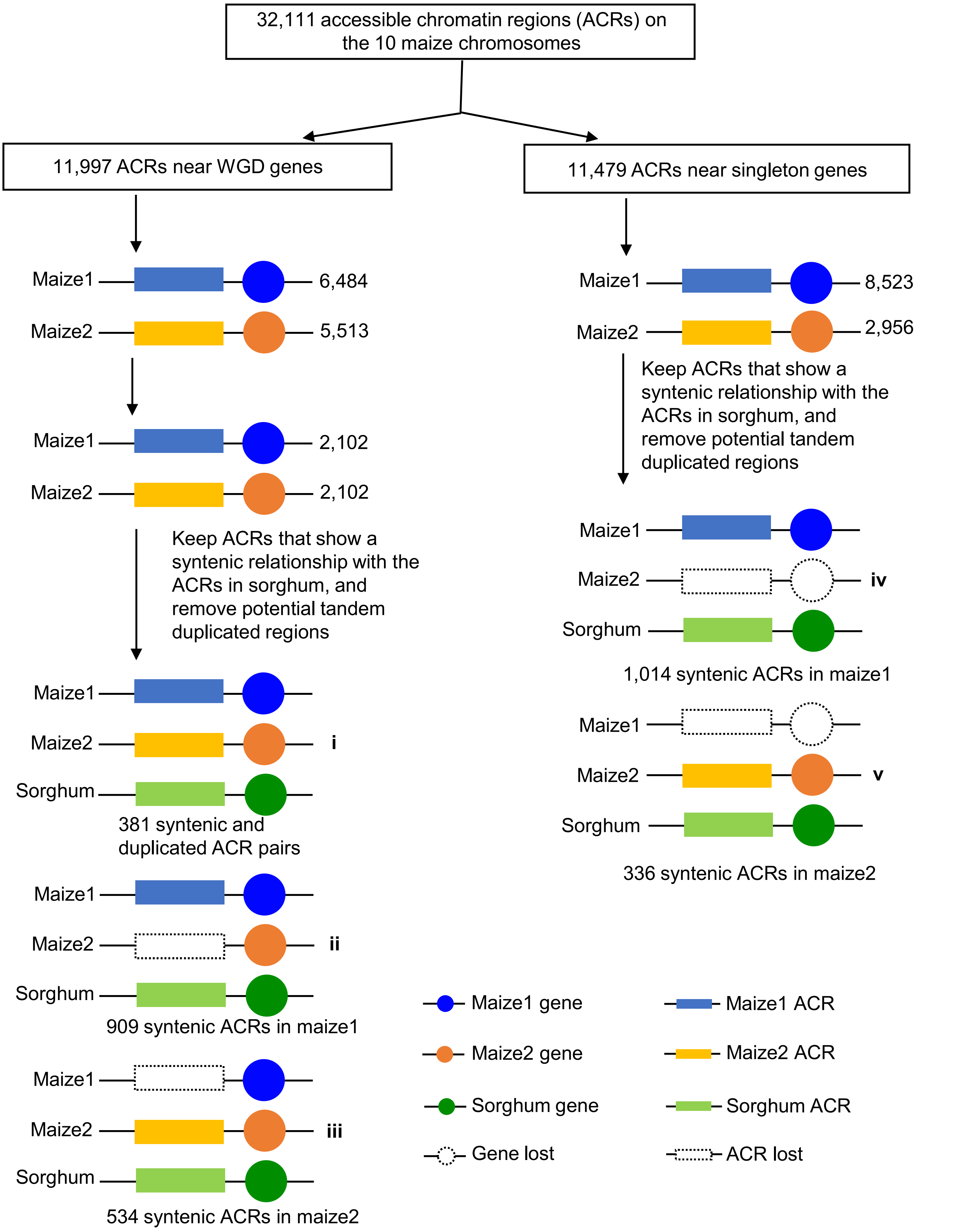

supplementary fig. S13. Bioinformatics pipeline to analyze syntenic accessible chromatin regions (ACRs).

Rectangles represent ACRs, and circles indicate WGD genes. Dotted rectangles and circles indicate lost ACRs or genes. WGD, whole genome duplication.

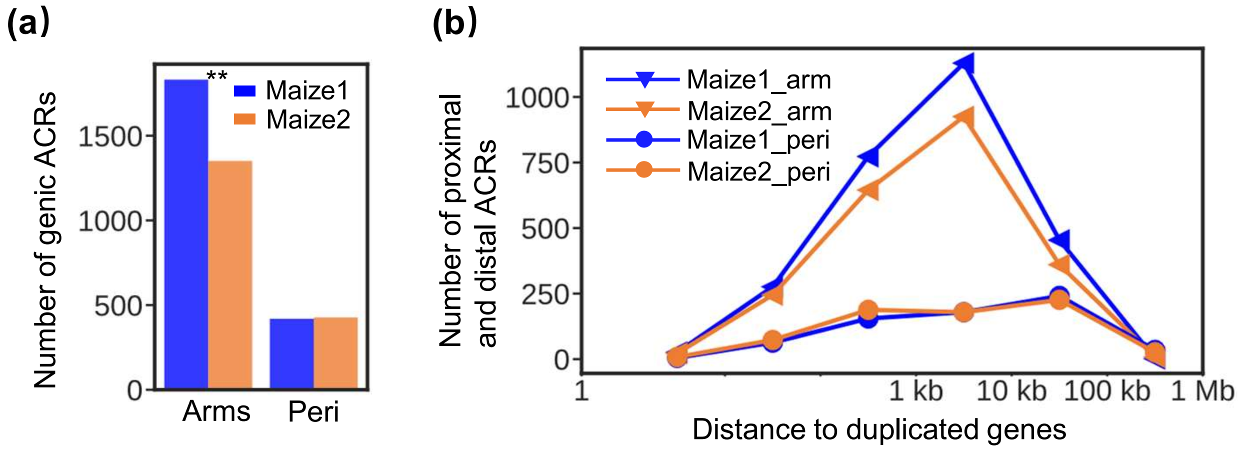

supplementary fig. S14. No significant differences in the numbers of accessible chromatin regions (ACRs) between maize1 and maize2 in pericentromeric regions from the ear tissue.
(a) Numbers of genic ACRs in chromosomal arms (arms) and in pericentromeric regions (peri). The statistical analysis was conducted using χ^2 test. **, *P* < 0.01.
(b) Numbers of proximal and distal ACRs and their distances to nearest WGD genes.

Arms, chromosomal arms; peri, pericentromeric regions.

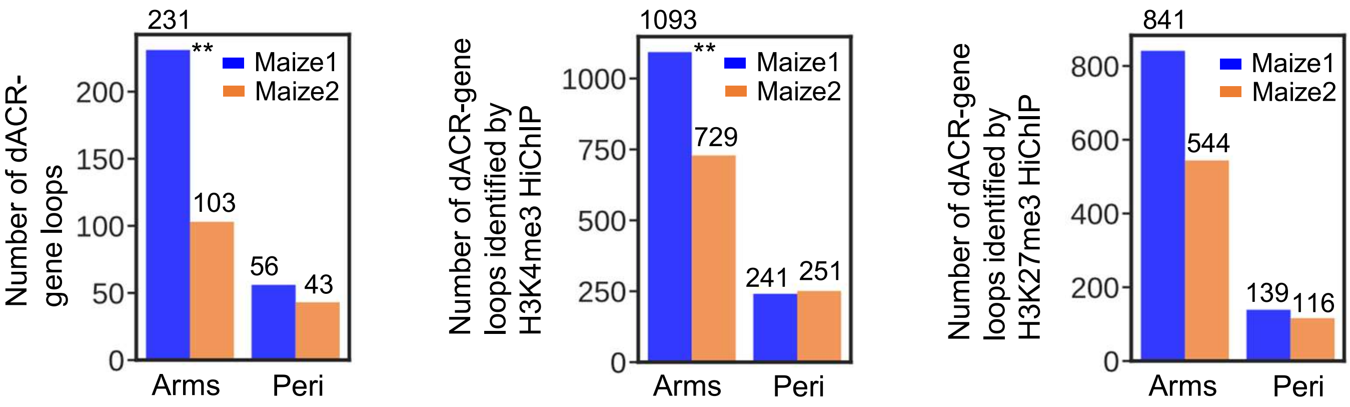

supplementary fig. S15. No significant differences in dACR-gene interactions between maize1 and maize2 in pericentromeric regions.

The statistical analysis was conducted using χ^2 test. **, $P < 0.01$. Arms, chromosomal arms; peri, pericentromeric regions.

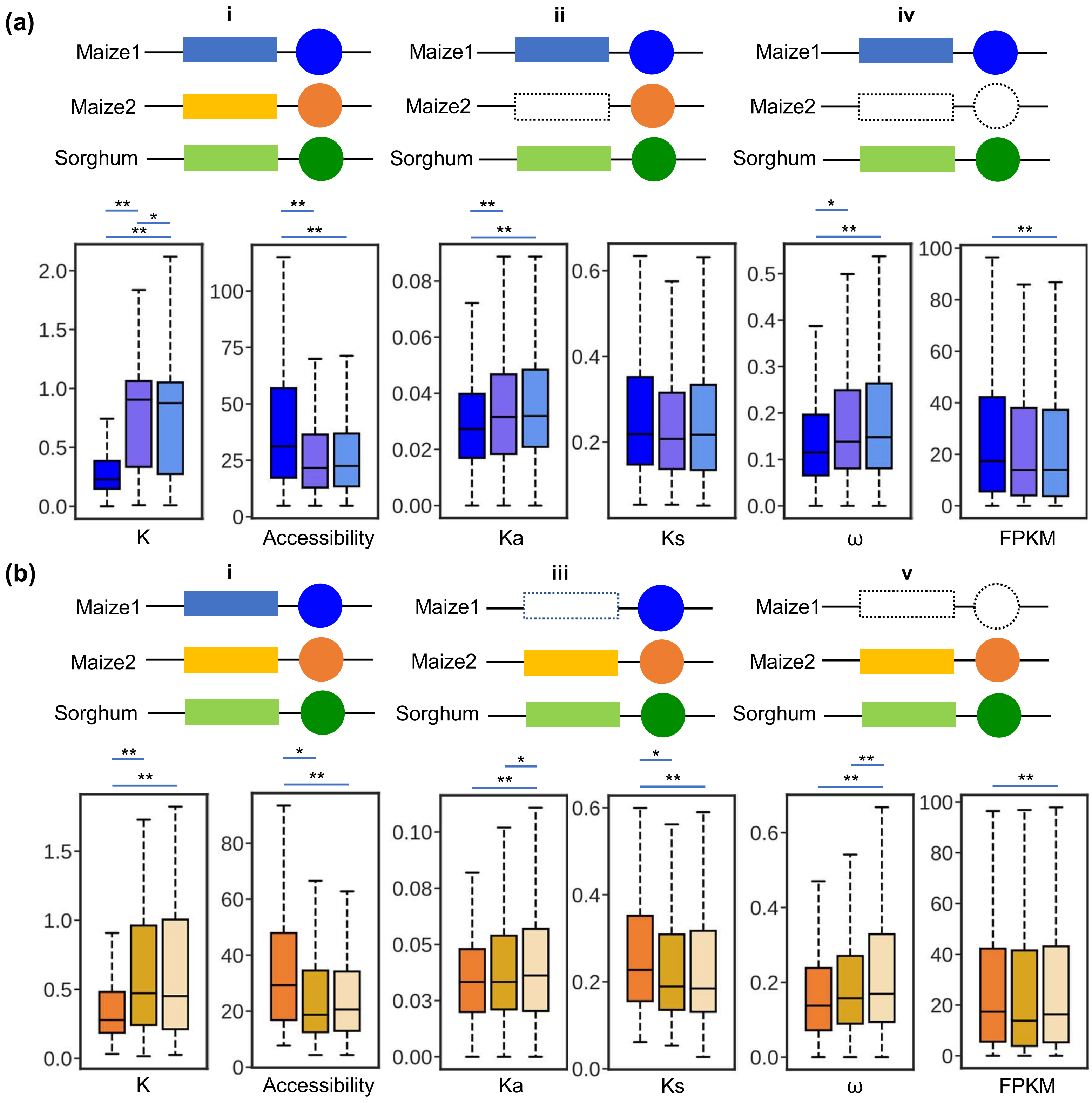

supplementary fig. S16. Comparisons of accessible chromatin regions (ACRs) and genes within each subgenome. **(a)** Duplicated versus singleton ACRs, and their flanking WGD genes versus singletons in maize1. **(b)** Duplicated versus singleton ACRs, and their flanking WGD genes versus singletons in maize2.

Evolutionary distances (K) and chromatin accessibilities of duplicated and singleton ACRs, and evolutionary distances (Ka, Ks, and ω) and expression values (FPKM, fragments per kilobase of exon per million mapped fragments) of WGD genes and singletons near these ACRs were compared in three different categories (i, iv, and v) described in supplementary fig. S13. WGD, whole genome duplication.

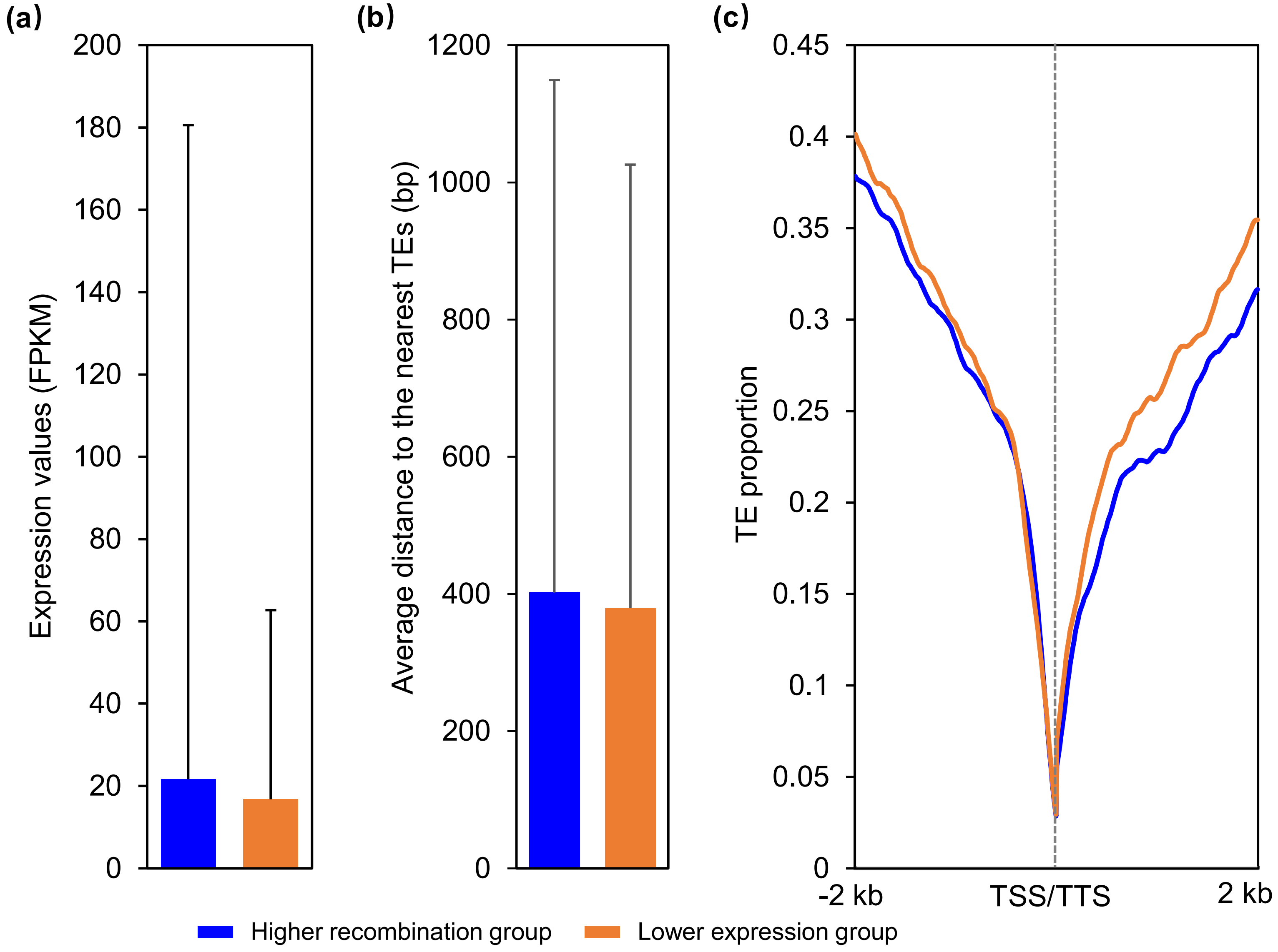

supplementary fig. S17. Comparison of WGD genes in the higher and lower recombination groups.
(a) Expression values. FPKM, fragments per kilobase of exon per million mapped fragments.
(b) Average distance to the nearest transposable elements (TEs).
(c) The abundance of TEs in the 2 kb upstream and downstream of the duplicated genes.

Higher-recombination genes are those whose recombination rates (cM/Mb) are at least 2-fold higher than their duplicated counterparts, which were classified into the lower recombination group. A total of 2,718 duplicated gene pairs were included in this analysis. WGD, whole genome duplication.

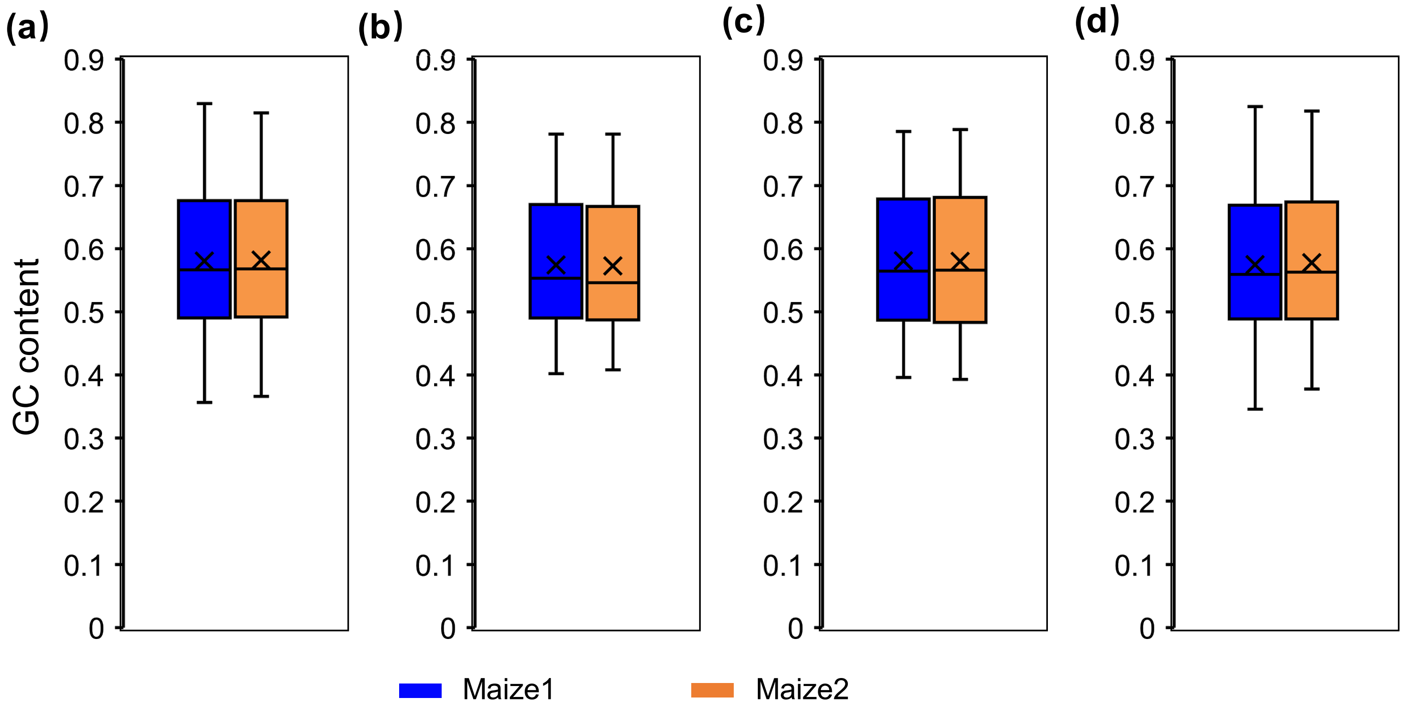

supplementary fig. S18. No significant difference with respect to GC content between maize1 and maize2.
(a) Both homoeologous genes in chromosome arms (M1_arm;M2_arm: 2,877 gene pairs).
(b) Both homoeologous genes in pericentromeric regions (M1_peri;M2_peri: 429 gene pairs).
(c) Maize1 genes in chromosome arms, and maize2 genes in pericentromeric regions (M1_arm;M2_peri: 720 gene pairs).
(d) Maize1 genes in pericentromeric regions, and maize2 genes in chromosome arms (M1_peri;M2_arm: 552 gene pairs).
